# Inhibition of multiple defense responsive pathways by CaWRKY70 transcription factor promotes susceptibility in chickpea under *Fusarium oxysporum* stress condition

**DOI:** 10.1186/s12870-020-02527-9

**Published:** 2020-07-06

**Authors:** Joydeep Chakraborty, Senjuti Sen, Prithwi Ghosh, Akansha Jain, Sampa Das

**Affiliations:** 1 Present Address: Division of Plant Biology, Bose Institute, Centenary Campus, P-1/12, CIT Scheme-VIIM, Kankurgachi, Kolkata, West Bengal 700054 India; 2 Present Address: Institute of Biological Chemistry, Washington State University, Pullman, Washington, USA

**Keywords:** Immune response, Protein-protein interaction, Reactive oxygen species (ROS), R-protein signaling, Systemic acquired resistance (SAR), Transcriptional regulation

## Abstract

**Background:**

Suppression and activation of plant defense genes is comprehensively regulated by WRKY family transcription factors. Chickpea, the non-model crop legume suffers from wilt caused by *Fusarium oxysporum* f. sp. *ciceri* Race1 (Foc1), defense response mechanisms of which are poorly understood. Here, we attempted to show interaction between WRKY70 and several downstream signaling components involved in susceptibility/resistance response in chickpea upon challenge with Foc1.

**Results:**

In the present study, we found *Cicer arietinum* L. WRKY70 (CaWRKY70) negatively governs multiple defense responsive pathways, including Systemic Acquired Resistance (SAR) activation in chickpea upon Foc1 infection. CaWRKY70 is found to be significantly accumulated at shoot tissues of susceptible (JG62) chickpea under Foc1 stress and salicylic acid (SA) application. *CaWRKY70* overexpression promotes susceptibility in resistant chickpea (WR315) plants to Foc1 infection. Transgenic plants upon Foc1 inoculation demonstrated suppression of not only endogenous SA concentrations but expression of genes involved in SA signaling. *CaWRKY70* overexpressing chickpea roots exhibited higher ion-leakage and Foc1 biomass accumulation compared to control transgenic (VC) plants. CaWRKY70 overexpression suppresses H_2_O_2_ production and resultant reactive oxygen species (ROS) induced cell death in Foc1 infected chickpea roots, stem and leaves. Being the nuclear targeted protein, CaWRKY70 suppresses CaMPK9-CaWRKY40 signaling in chickpea through its direct and indirect negative regulatory activities. Protein-protein interaction study revealed CaWRKY70 and CaRPP2-like CC-NB-ARC-LRR protein suppresses hyper-immune signaling in chickpea. Together, our study provides novel insights into mechanisms of suppression of the multiple defense signaling components in chickpea by CaWRKY70 under Foc1 stress.

**Conclusion:**

CaWRKY70 mediated defense suppression unveils networking between several immune signaling events negatively affecting downstream resistance mechanisms in chickpea under Foc1 stress.

## Background

Plant defense against pathogens are rapidly conveyed through cell surface receptors or by the intracellular immune receptors. Cell surface receptors usually recognize specific pathogen or microbe associated molecular patterns (i.e., PAMPs or MAMPs) and elicits Pattern Triggered Immunity (PTI). By contrast, intracellular receptors bind PTI suppressing effector proteins released by the pathogens which induce strong immune response, known as Effector Triggered Immunity (ETI) [1, 2]. WRKY transcription factors (TFs) are indispensable regulators of both PTI and ETI to wide variety of pathogens. The members of large multigene family transcription factor comprise WRKY domain (WRKYGQK) and zinc finger motif (CX4-7CX22-23HXH/C) that binds at TGAC core of W-box containing DNA [3, 4]. There are 74 WRKY family members present in *Arabidopsis thaliana*, which have been classified into three major groups (I, II and III) based on the number and position of WRKY domains and features of the zinc finger motif [5].

Transcriptional regulation of plant defense related gene expression by WRKY proteins are crucial to enable the induction of host immunity. Binding of WRKY70 TF at promoters of SA and JA signaling pathway genes, such as *NPR1*, *PR2*, *PR10*, *VSP1* and *VSP2* are associated with positive regulation of plant defense signaling [6–9]. *AtWRKY33* overexpression leads to enhanced resistance against necrotrophic fungal pathogens, *Botrytis cinerea* and *Alternaria brassicicola*, although, plants showed susceptibility to *Pseudomonas syringae* infection [10]. WRKY28 and WRKY46 play co-transcriptional regulators of *ISOCHORISMATE SYNTHASE1* (*ICS1*) gene expression and SA biosynthesis which mount defense response against biotrophic pathogens [11]. SA accumulation severely affects both PTI and ETI [12, 13]. Activation of Systemic Acquired Resistance (SAR) in the pathogen free distal tissues is also dependent on SA accumulation and signaling that trigger resistance against a large variety of pathogens, including viruses, bacteria and fungi [14–16]. In *Arabidopsis*, *SA INDUCTION-DEFICIENT2* (*SID2*), *ENHANCED DISEASE SUSCEPTIBILITY5* (*EDS5*), and *NONEXPRESSOR OF PR GENES1* (*NPR1*) control SA production and signaling on pathogen challenge [17]. *SID2* encodes an isochorismate synthase enzyme that converts chorismate to isochorismate [18]. Pathogen induced expression of *SID2,* and concomitant SA accumulation is regulated by *SYSTEMIC ACQUIRED RESISTANCE DEFICIENT 1* (*SARD1*) [19]. SARD1 positively regulates *ICS1* gene expression that promotes pathogen-inducible SA accumulation in *Arabidopsis* [19, 20]. AtWRKY70 binds at promoter and inhibits *SARD1* expression, which lowers the endogenous SA levels [20]. AtWRKY70 also functions as transcriptional regulator of JA/ ET induced gene expression and Induced Systemic Resistance (ISR) triggered by *Bacillus cereus* AR156 [21]. The apparent positive or negative effects of AtWRKY70 on transcription may thus provide the mechanistic basis for regulation of SA induced defense gene expression during local and systemic resistance in *Arabidopsis*.

ROS are the primary inducer for plant defense signaling that can trigger activation of mitogen activated protein kinase (MPK) cascade during plant-pathogen interplay [22, 23]. ROS production leads to upregulation of genes involved in SA- and JA/ ET- signaling pathway [24]. Furthermore, SA and ROS together play crucial roles in hypersensitive response (HR) triggered cell death signaling during SAR development in *Arabidopsis* [25]. Respiratory Burst Oxidase Homologs (RBOHs), a plasma membrane bound NADPH oxidase contribute ROS production in *Arabidopsis thaliana* and *Nicotiana benthamiana* [26, 27]. WRKYs are the transcriptional regulator of ROS production in these plants. WRKYs regulate the expression of *AtRBOHD* and *AtRBOHF* that mediate ETI-induced ROS bursts [26]. WRKY8 triggers *NbRBOHB* expression and HR induced cell death in *N. benthamiana* [27]. Treatment of *Arabidopsis* leaves with H_2_O_2_, a primary ROS candidate also upregulates the expression of many *WRKY* genes [28]. Thus, *WRKY* genes expression and ROS production are coordinately regulated at transcriptional level that prompts the activation of multiple defense signaling pathways like, hormonal crosstalk, ROS signaling, MAPK signaling, and HR associated cell death.

HR develops only when an appropriate Avr (avirulent) protein interacts with its cognate R (resistance) protein *in planta* [29, 30]. Effector proteins often target WRKYs in order to manipulate plant immunity. It is a well-known fact that WRKYs and R proteins serve common regulators of resistance signaling pathways to several plant-pathogen interactions. *Arabidopsis* Resistance to *Ralstonia solanacearum* 1 (RRS1) carries an extra integrated WRKY domain at its C-terminal end. This type of extended WRKY module perceives PopP2 effector protein and protects acetylation of other WRKYs upon instigating strong immune responses to the bacterial pathogen *R. solanacearum* [31]. It is important that RRS1 with its single WRKY domain can induce transcriptional reprogramming during ETI. WRKY70 also contributes to Recognition of *Peronospora Parasitica* 4 (RPP4)-mediated resistance against *Hyaloperonospora parasitica* [32]. Our recent study has established that Foc1 resistance in chickpea is dependent on the interaction between RPP2-like CC-NB-ARC-LRR protein and CaWRKY64 [30].

The present study has been focussed on chickpea-*Fusarium* interaction since, a smaller number of reports are currently available on legume-fungus interactions and detailed molecular regulations are undoubtedly obscured. Chickpea (*Cicer arietinum* L.) is the world’s third most important pulse crop and a rich source of plant-derived edible protein. Chickpea production has been severely affected by wilt-causing hemi-biotrophic fungus *Fusarium oxysporum* f. sp. *ciceri* Race1 (Foc1) [33]. Amongst the eight different pathogenic races of *Fusarium oxysporum* f. sp. *ciceri*, Race1 is known to have cosmopolitan distribution causing significant yield losses. Foc1 infection accounting 10–15% annual crop loss and reaches 90–100% during favourable season [34, 35]. Foc1 invades chickpea through roots and grows to shoots where it colonizes the xylem vessels at root-stem interface region. Increasing fungal biomass blocks water supply to the aerial shoots, which results in massive vascular wilting [34, 35]. Foc1 resistance in chickpea is hard to achieve by usual breeding approaches due to limited genetic resources and elevated autogamy [36]. We used wilt-susceptible JG62 and wilt-resistant WR315 chickpea accessions to unveil the immunomodulatory role of CaWRKY70 protein on Foc1 infection [36, 37]. Our study shows that CaWRKY70 transcription factor promotes susceptibility in chickpea upon Foc1 infection. CaWRKY70 inhibits SA concentrations and signaling in non-inoculated distal shoot tissues of transgenic chickpea. Transcripts measurement data suggests CaWRKY70 functions as negative regulator for subsets of immune-responsive genes that control defense responses in chickpea, including *PR* genes. CaWRKY70 also suppresses endogenous ROS levels and R-protein induced ectopic cell death. Together, we establish that CaWRKY70 negatively impacts defense signaling and SAR development in chickpea under Foc1 stress.

## Results

### Systemic expression pattern of *Cicer arietinum* L. WRKY70 (*Ca*WRKY70) under SA induction and Foc1 infection

**Fig. 1:**
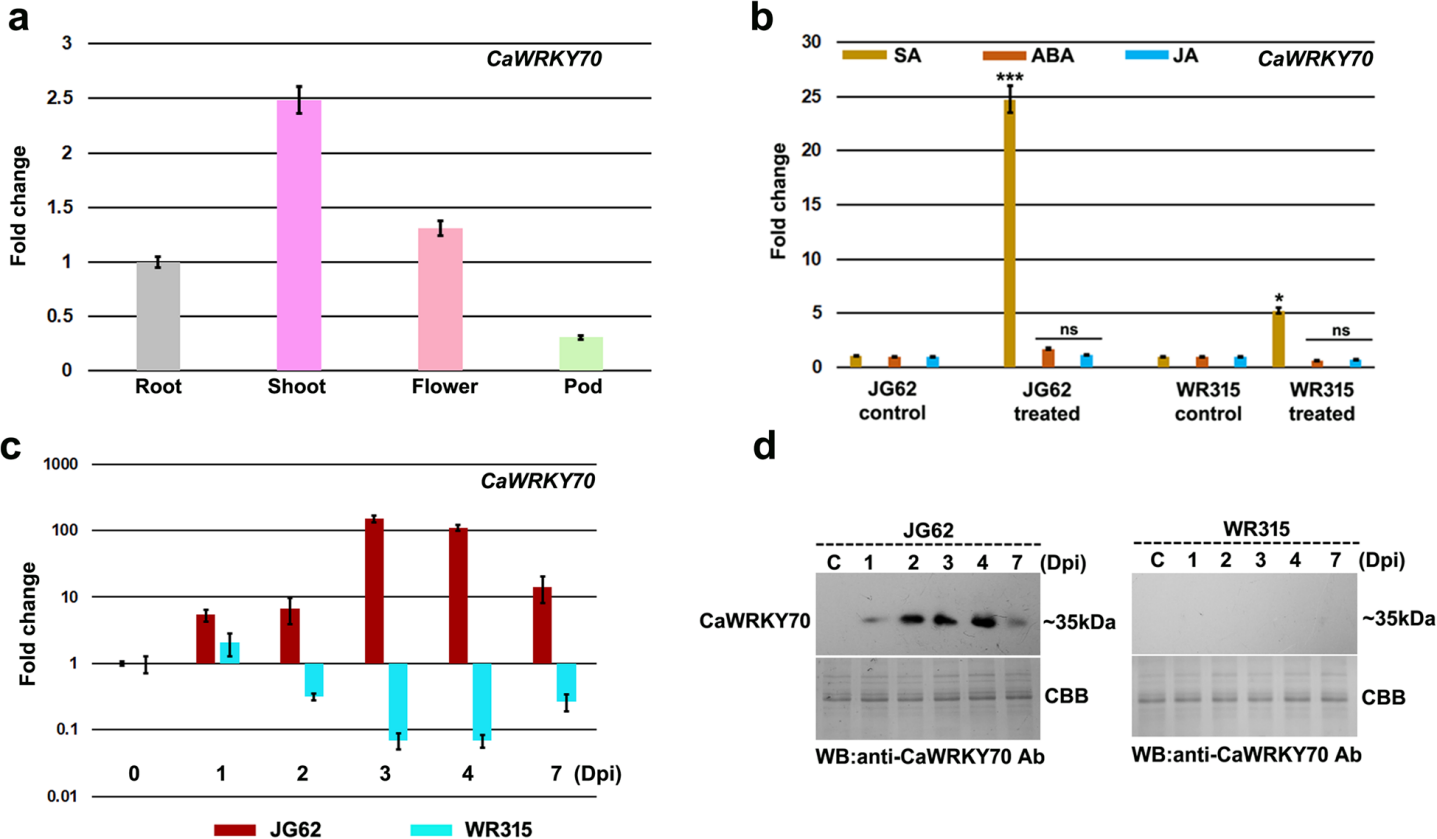
Expression pattern of CaWRKY70 transcript and protein in chickpea. **a** Organ specific expression of *CaWRKY70* in chickpea root, shoot, flower and pod tissues, respectively. *CaWRKY70* mRNA levels in different organs were measured using *CaGAPDH* as internal control. Error bars represent mean ± SD of three independent biological replicates. **b***CaWRKY70* transcript levels after SA, ABA and JA treatments in susceptible JG62 and resistant WR315 chickpea shoots. *CaGAPDH* was used as internal control. Error bars represent ±SD (*n* = 3). Asterisks (*) indicate values that differ significantly from control treatment (0 dpi) as determined by Student’s *t* test (*P ≤ 0.05 and ***P ≤ 0.001). NS denotes not significant. **c** Normalized fold induction of *CaWRKY70* transcript at shoot tissues of susceptible JG62 and resistant WR315 chickpea upon Foc1 infection. *CaWRKY70* fold change values at different time-points are normalized against 0 dpi in JG62 and those in WR315, respectively. *CaGAPDH* expression was used as the reference control. Error bars represent ±SD values of three biological replicates. **d** CaWRKY70 protein accumulation was determined by western blotting with anti-CaWRKY70 antibody. Coomassie Brilliant Blue (CBB) stained gels serve loading control

*CaWRKY70* expression in different chickpea tissues were determined by quantitative real-time PCR (qRT-PCR) analysis. *CaWRKY70* mRNA expression was detected in all tissues including root, shoot, flower and pod. *CaWRKY70* transcript accumulation was higher at shoot tissues compared to flower, root and pod in non-infected plants. *CaWRKY70* expression was detected to be ~ 2.5-fold higher at shoot tissues than the roots (Fig. 1a). Differential salicylic acid (SA) accumulation induces SAR activation at distal shoot tissues of susceptible and resistant chickpea plants upon Foc1 infection [38]. To investigate whether SA signaling influences *CaWRKY70* expression in chickpea, we measured *CaWRKY70* transcript abundance in shoot tissues of susceptible and resistant chickpea at 6 h of SA treatment. Result suggests that SA treatment promotes significantly higher accumulation of *CaWRKY70* transcript at shoot tissues of susceptible chickpea over control treatment. However, resistant plant shows less induction of *CaWRKY70* transcript upon exogenous SA application (Fig. 1b). Other inducers like, ABA and JA failed to stimulate *CaWRKY70* expression neither in susceptible nor in the resistant chickpea shoots. Therefore, it may be suggested that differential expression of *CaWRKY70* in chickpea is mediated through SA response under Foc1 infection. *Arabidopsis* WRKY70 is an important WRKY member that has been shown to regulate SAR activation against biotrophic pathogens [9, 39]. To ascertain whether *CaWRKY70* is associated with the systemic defense responses of chickpea, we sought to determine its mRNA expression at shoot tissues under control treatment and Foc1 infection. Susceptible and resistant chickpea plants subjected to Foc1 infection at 1, 2, 3, 4 and 7 days were used for RNA isolation, cDNA preparation and qRT-PCR analyses. *CaWRKY70* expression at different time-points as compared to the 0 dpi control treatment in susceptible JG62 and resistant WR315 were plotted (Fig. 1c). *CaWRKY70* fold change levels were normalized to a value of 1 at 0 dpi in JG62 and WR315, respectively. *CaWRKY70* transcript was found to be induced at shoot tissues of susceptible chickpea on challenge with Foc1. On the contrary, resistant chickpea plants were unable to stimulate *CaWRKY70* expression at shoot tissues under Foc1 stress. Time-dependent data revealed that *CaWRKY70* transcript was found to be ~ 100-fold upregulated at 3 dpi in susceptible chickpea shoots over control treatment (Fig. 1c). However, the inclusion of mock treatment to each time point would have been useful to determine the developmental stage specific *CaWRKY70* expression in both susceptible and resistant chickpea upon Foc1 infected conditions. To investigate CaWRKY70 protein accumulation, total proteins were extracted from control and Foc1 infected susceptible and resistant chickpea shoot tissues. Next, immunoblotting experiment was performed using anti-CaWRKY70 polyclonal antibody. Result shows induction of ~ 35 kDa CaWRKY70 protein band in non-inoculated systemic shoot tissues of susceptible plants upon Foc1 infection, whereas resistant plants failed to stimulate such protein accumulation (Fig. 1d). The protein level was higher at shoot tissues of susceptible plant at 4 dpi on Foc1 challenge, suggesting systemic accumulation of CaWRKY70 in susceptible chickpea.

### CaWRKY70 is nuclear targeted protein

To test subcellular localization of CaWRKY70 protein, we fused yellow fluorescence protein (YFP) at its C-terminus. CaWRKY70 protein fused to YFP was expressed in onion epidermal cells by *Agrobacterium* mediated transient transformation. Contrarily, an empty construct expressing control YFP was tested. Onion epidermal cells were stained with nuclear marker 4′, 6-diamidino-2-phenylindole (DAPI). Confocal microscopic analyses showed that CaWRKY70-YFP was localized in nucleus. In contrast, localization of control YFP protein was observed throughout the cells (Additional file 1: Figure S1). Result also revealed that blue color fluorescence of DAPI-stained nuclei is overlapped with yellow color fluorescence of YFP, thereby, confirming nuclear localization of CaWRKY70.

### *CaWRKY70* overexpression triggers susceptibility in chickpea to Foc1

**Fig. 2:**
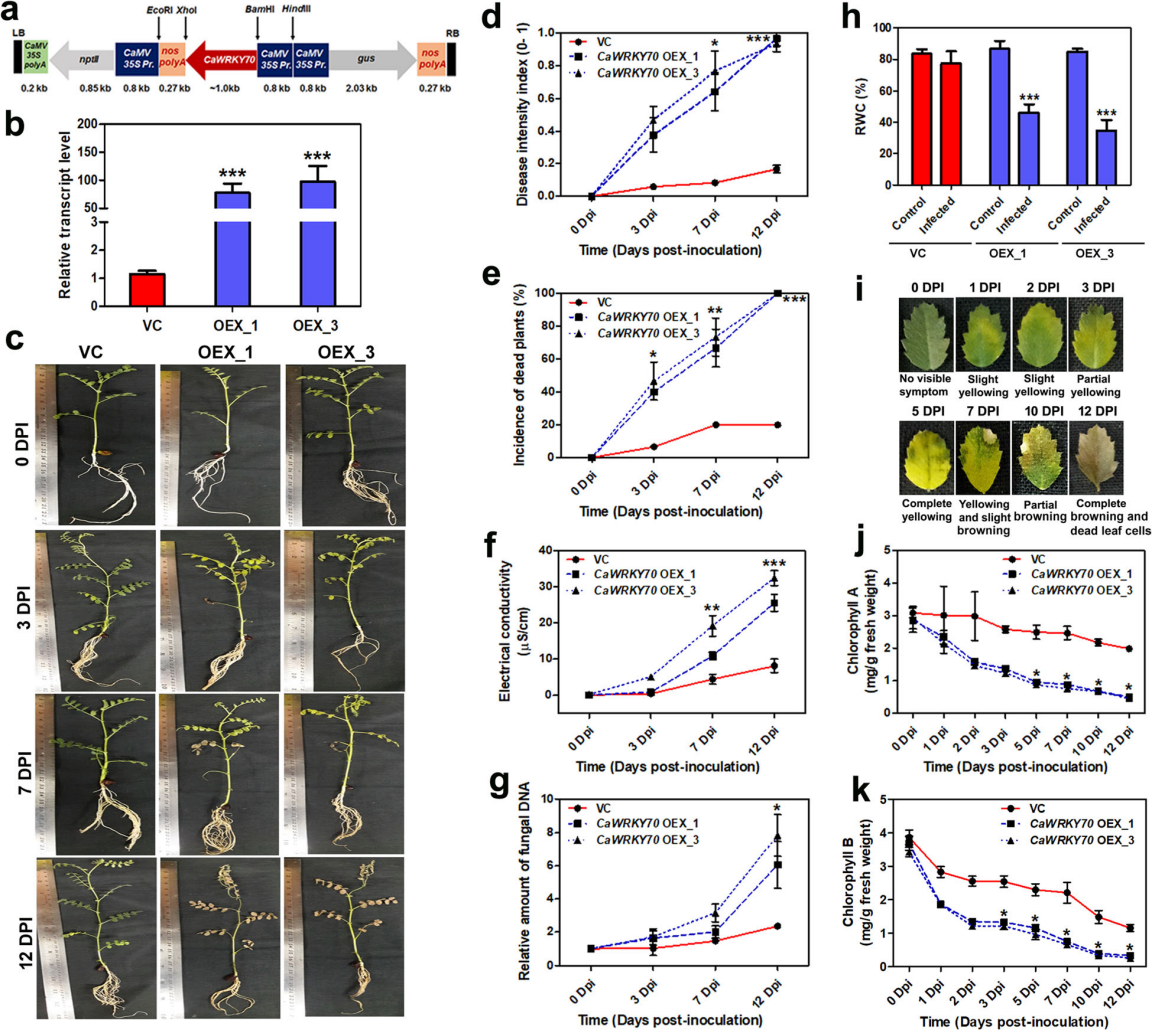
*CaWRKY70* overexpression induces susceptibility in chickpea under Foc1 infection. **a** Diagram of gene construct used for *CaWRKY70* overexpression in resistant chickpea. **b** qRT-PCR determination of *CaWRKY70* mRNA level in control transgenic (VC) and overexpressing chickpea. Error bars indicate ±SD of three independent biological samples. Data was normalized to *CaGAPDH*. Fold change values relative to control vector transformed plants. **c** Development of disease symptom in control transgenic and *CaWRKY70* overexpressing chickpea. Disease phenotype of transgenic plants were photographed under control treatment (0 dpi) and Foc1infection at 3, 7 and 12 dpi, respectively. **d** Assessment of disease intensity index in control transgenic and *CaWRKY70* overexpressing chickpea under control treatment (0 dpi) and Foc1infection. Chickpea plants grown in the soil-rite mixture were inoculated with Foc1. **e** Effect of Foc1 infection on the control transgenic and *CaWRKY70* overexpressing chickpea based on incidence of dead or wilted plants. In **d** and **e**, each data point represents mean values of three pots with five plants per pot. Error bars indicate ±SD of three independent biological samples. **f** Electrolyte leakage in control transgenic (VC) and *CaWRKY70* overexpressing chickpea roots under control treatment (0 dpi) and Foc1 infection. Each time point represents ±SD of three biological replicates. *P ≤ 0.05 and **P ≤ 0.01 indicate values show significant differences between control transgenic (VC) and *CaWRKY70* overexpressing chickpea as determined by Student’s *t* test. **g** Quantitation of Foc1 5.8S rDNA at root tissues of control transgenic (VC) and *CaWRKY70* overexpressing chickpea plants by real-time PCR. Chickpea DNA amount was normalized by *CaGAPDH* expression. Each bar represents mean ± SD of three independent biological replicates and fold change is relative to control treatment (0 dpi). **P ≤ 0.01 indicate mean values significantly different from control vector transformed plant determined by Student’s *t* test. **h** Relative water content percent (RWC%) in control transgenic and *CaWRKY70* overexpressing chickpea under control treatment and Foc1 infection. Asterisks (*) indicate significant difference at ***P ≤ 0.001 by one-way ANOVA followed by multiple comparison of means using tukey’s post-hoc test. **i** Disease symptoms in chickpea leaves for Foc1 varied from yellowing to browning or cell death at different infection time-points. **j** Chlorophyll A and (**k**) Chlorophyll B content in control transgenic and *CaWRKY70* overexpressing chickpea leaves at different infection time-points. In **j** and **k**, error bars represent ±SD (n = 3). *P ≤ 0.05 indicate mean values are significantly different from control vector transformed plants as determined by Student’s *t* test

To examine the possible role of *CaWRKY70* in defense regulation against Foc1, full-length *CaWRKY70* gene was isolated from Foc1 treated shoot tissues of susceptible chickpea cDNA sample. Chimeric *CaWRKY70* gene construct was prepared using pCAMBIA2301 vector and positive clones were selected by PCR analyses (Additional file 1: Figure S2). *CaWRKY70* gene containing plasmids were delivered into resistant chickpea genome via *Agrobacterium* mediated transformation and transgenic chickpea plants were established (Fig. 2a, b). Two independent *CaWRKY70* overexpressing chickpea lines i.e., OEX1, and OEX3 of T_2_ generation were obtained from primary transformants. Real-time PCR analysis detected that overexpressing plants exhibit significantly higher expression of *CaWRKY70* transcript than control vector transformed plants, where lowest expression of *CaWRKY70* mRNA was noted (Fig. 2b). To compare disease symptoms on control transgenic and *CaWRKY70* overexpressing chickpea, we treated plants with Foc1 for 0, 3, 7 and 12 days, respectively. Result shows that *CaWRKY70* overexpressing chickpea plants exhibit enhanced susceptibility to Foc1 infection in comparison to the control transgenic plants. Disease symptoms were much pronounced in overexpressing chickpea plants at 12 dpi with Foc1 (Fig. 2c). Foliar symptom that developed on control transgenic and *CaWRKY70* overexpressing chickpea upon interaction with Foc1 was used to measure the disease intensity index. Based on incidence of infected plants and foliar symptoms, *CaWRKY70* overexpressing chickpea was found to be highly susceptible to Foc1 than control transgenics. Disease symptom started developing at 3 dpi, progressed at higher rates in *CaWRKY70* overexpressing chickpea resulting in 100% plants with vascular wilt developed at 12 dpi (Fig. 2d). *CaWRKY70* transgenic plants developed highly susceptible reaction to Foc1 reaching 100% incidence of dead plants at 12 dpi. In contrast, control transgenic plants demonstrated less severe reaction at 12 dpi with only 20% of the plant’s dead (Fig. 2e). Foc1 infection progressively enhances cell-death induced ion-leakage at root tissues of control transgenic and *CaWRKY70* overexpressing chickpea. Time-dependent analyses show significant difference in Foc1 induced electrolyte leakage in control transgenic and *CaWRKY70* overexpressing chickpea with higher leakage of ions in overexpressing chickpea roots and least in control transgenic plants. The conductivity was found to be significantly increased in *CaWRKY70* overexpressing root at 12 dpi of Foc1 infection than control transgenics (Fig. 2f). Amount of Foc1 biomass in root tissues of *CaWRKY70* overexpressing chickpea appears to be significantly higher than control transgenic. The relative accumulation of Foc1 5.8S rDNA was ~ 2.0-fold and ~ 7.0-fold at root tissues of control transgenic and *CaWRKY70* overexpressing chickpea, respectively, under Foc1 infection (Fig. 2g). Relative water content (RWC) was determined to compare percentage amount of water restored within the plant body of control transgenic and *CaWRKY70* overexpressing chickpea under control condition and Foc1 infection. Result shows that control transgenic plants retain ~ 77% RWC upon Foc1 infection whereas, the value was markedly reduced in *CaWRKY70* overexpressing chickpea plants under Foc1 stress i.e., ~ 46% and ~ 34% RWC (Fig. 2h). Foc1 inoculated chickpea plants show chlorosis of leaves accompanied by yellowing to browning or cell death (Fig. 2i). Decrease in chlorophyll A and chlorophyll B content was measured using leaves of control transgenic and *CaWRKY70* overexpressing chickpea in time dependent manner. Control transgenic plants retain significantly higher chlorophyll A and chlorophyll B content than overexpressing chickpea on Foc1 stress (Fig. 2j, k). Nevertheless, loss of total chlorophyll content in chickpea leaves might be a secondary effect of Foc1 infection. Taken together, our results confirm that *CaWRKY70* promotes susceptibility in chickpea to Foc1.

### CaWRKY70 reduces ROS accumulation and cell death in chickpea

**Fig. 3:**
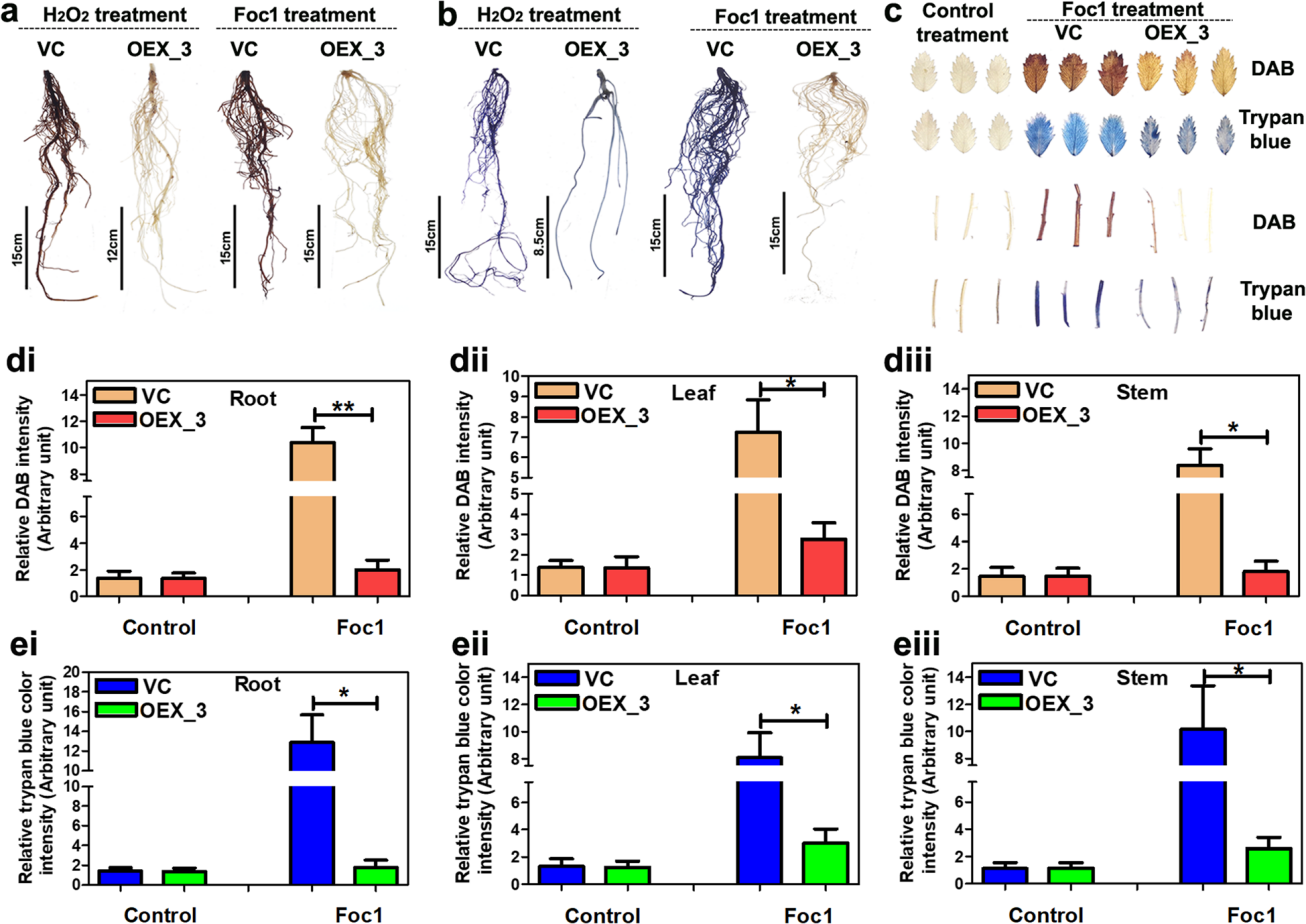
H_2_O_2_ accumulation and cell death in different chickpea organs of control transgenic and *CaWRKY70* overexpressing plants upon Foc1 infection. **a**, **b** DAB and trypan blue staining of chickpea roots under H_2_O_2_ treatment and Foc1 infection. **c** DAB and trypan blue colouration of leaves and stem portions from control transgenic and *CaWRKY70* overexpressing chickpea plants. In **a**, **b** and **c**, chickpea plants were infected with Foc1 for 7 days. 10 mM H_2_O_2_ treatment was carried out for 30 min using chickpea roots. **d** Quantification of relative DAB staining activities in control transgenic and *CaWRKY70* overexpressing chickpea roots (i), leaves (ii) and stem (iii) under control treatment Foc1 infection. (**e**) Quantitation of relative trypan blue colour intensities in control transgenic and *CaWRKY70* overexpressing chickpea roots (i), leaves (ii) and stem (iii) under control treatment (0 dpi) and Foc1 stress. In **d** and **e**, error bars represent ±SD (n = 3). Asterisks (*) indicate values which are significantly different between control transgenic and *CaWRKY70* overexpressing chickpea plants as determined by Student’s *t* test (*P ≤ 0.05 and **P ≤ 0.01)

Histochemical DAB and trypan blue staining was performed to compare H_2_O_2_ accumulation and cell death between control transgenic and *CaWRKY70* overexpressing chickpea under Foc1 stress. Result demonstrates intense DAB and trypan blue colouration in Foc1 infected control transgenic roots (Fig. 3a, b). On the contrary, DAB and trypan blue staining was not observed at *CaWRKY70* overexpressing chickpea roots under Foc1 stress. H_2_O_2_ treatment was carried out as primary ROS inducer in transgenic chickpea roots which show intense DAB and trypan blue staining at roots of control transgenic plants. DAB colouration was not demonstrated by *CaWRKY70* overexpressing chickpea roots. However, faint trypan blue colour was retained by overexpressing plant roots only after H_2_O_2_ treatment (Fig. 3a, b). DAB and trypan blue staining was also performed using leaves and stem parts of the control transgenic and *CaWRKY70* overexpressing chickpea plants. Here, no such DAB or trypan blue staining was noted upon control treatment, however, Foc1 infection resulted in higher deposition of brownish DAB precipitates in leaves and stem tissues of control transgenic plants in comparison to *CaWRKY70* overexpressing chickpea (Fig. 3c). Similarly, strong trypan blue colouration was noted on leaves and stem portions of control transgenic plants than *CaWRKY70* overexpressing chickpea upon Foc1 infection (Fig. 3c). It is noteworthy that *CaWRKY70* overexpressing chickpea leaves and stem show mild histochemical DAB and trypan blue staining. We further measured DAB and trypan blue colour intensities of control transgenic and *CaWRKY70* overexpressing chickpea root, leaf and stem tissues. Quantitative measurements revealed significant reduction in the DAB and trypan blue intensities at roots, leaves and stem parts of *CaWRKY70* overexpressing chickpea compared to control transgenic plants under Foc1 stress (Fig. 3d, e). Although, control treatment does not exhibit such drastic changes in DAB and trypan blue intensities of empty vector transformed and *CaWRKY70* overexpressing chickpea plants. Therefore, it can be concluded that CaWRKY70 negatively affects ROS accumulation and cell death induction and thus promotes susceptibility in *CaWRKY70* overexpressing chickpea plants upon Foc1 infection.

### CaWRKY70 inhibits SA signaling in transgenic chickpea

**Fig. 4:**
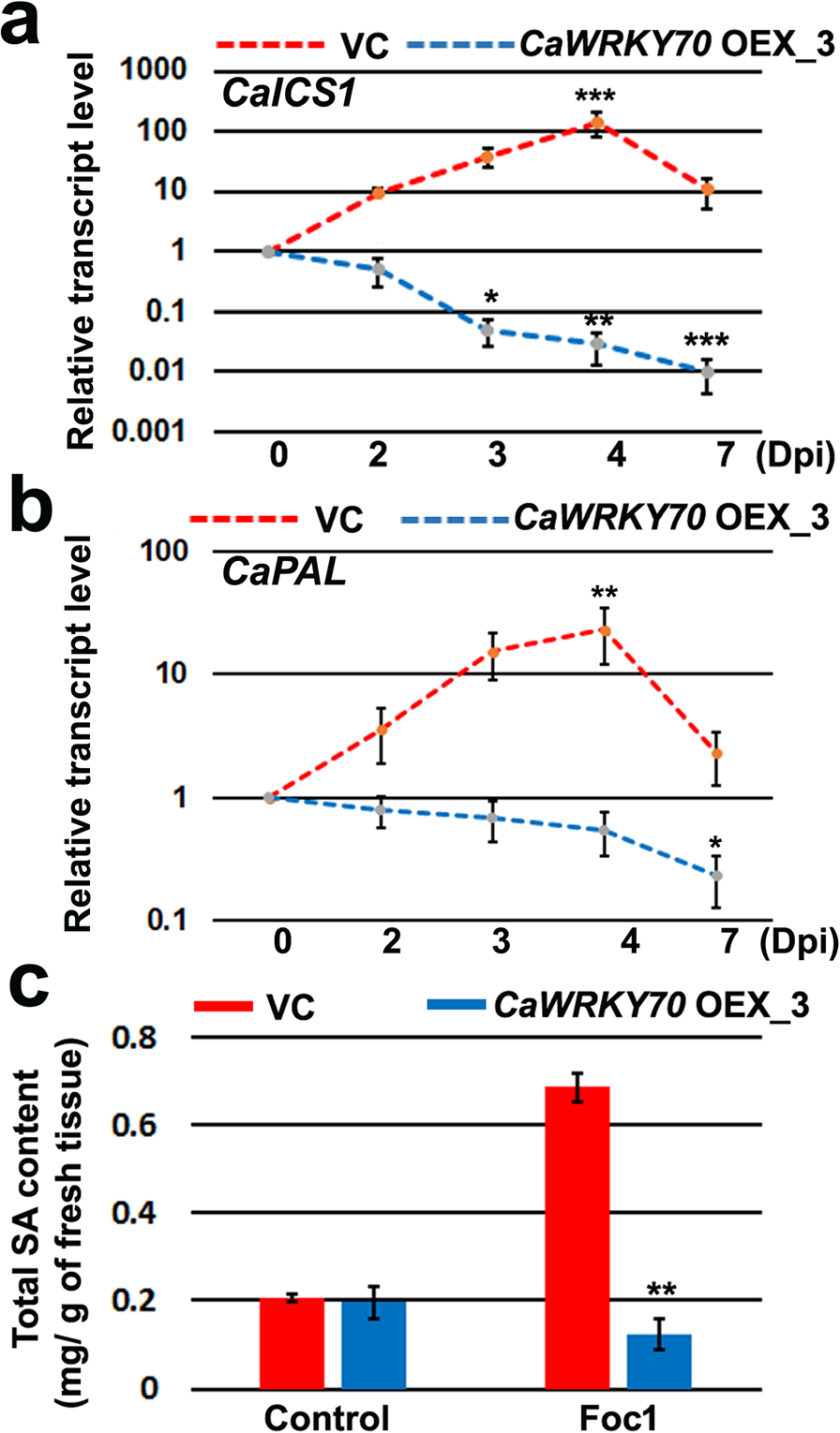
SA signaling gene expression and SA concentrations at shoot tissues transgenic chickpea under Foc1 stress. **a***CaICS1* (XM_004514070.3), (**b**) *CaPAL* (NM_001279177.2) transcripts level at shoot tissues of control transgenic (VC) and *CaWRKY70* overexpressing chickpea under Foc1 infection. *CaGAPDH* was used as internal control. In **a** and **b**, Error bars represent mean values ±SD (n = 3). Asterisks (*) indicate values that differ significantly from control treatment (0 dpi) as determined by Student’s *t* test (*P ≤ 0.05, **P ≤ 0.01 and ***P ≤ 0.001). (**c**) Total SA content at control transgenic (VC) and *CaWRKY70* overexpressing chickpea shoot tissues under control treatment (0 dpi) and Foc1 infected condition (7 dpi). Each bar represents mean ± SD of three independent biological samples. **P ≤ 0.01 indicates mean values showing significant difference between control transgenic and *CaWRKY70* overexpressing chickpea by Student’s *t* test

ICS1 and PAL contribute to the pathogen induced SA production through isochorismate and phenyl-propanoid pathways, respectively [18, 40]. In *CaWRKY70* overexpressing chickpea shoots, *CaICS1* and *CaPAL* transcripts were found to be downregulated after Foc1 infection at 2, 3, 4 and 7 days. Interestingly, pCAMBIA2301 vector transformed plant demonstrates mRNA induction upto 4 dpi and then downregulation at 7 dpi under Foc1 stress (Fig. 4a, b). SA concentrations were also significantly decreased at shoot tissues of *CaWRKY70* overexpressing chickpea in comparison to the control transgenic plants at 7 dpi with Foc1 (Fig. 4c). Result shows higher accumulation of SA in non-inoculated systemic shoot tissues of control vector transformed chickpea after Foc1 infection. It has been observed that control transgenic and *CaWRKY70* overexpressing chickpea exhibit basal accumulated level of SA production at shoot tissues under control treatment. Thus, CaWRKY70 reduces both SA biosynthesis genes expression and SA accumulation at shoot tissues of transgenic chickpea.

### Differential expression pattern of defense related transcripts in vector transgenic and *CaWRKY70* overexpressing chickpea under Foc1 infection

**Fig. 5:**
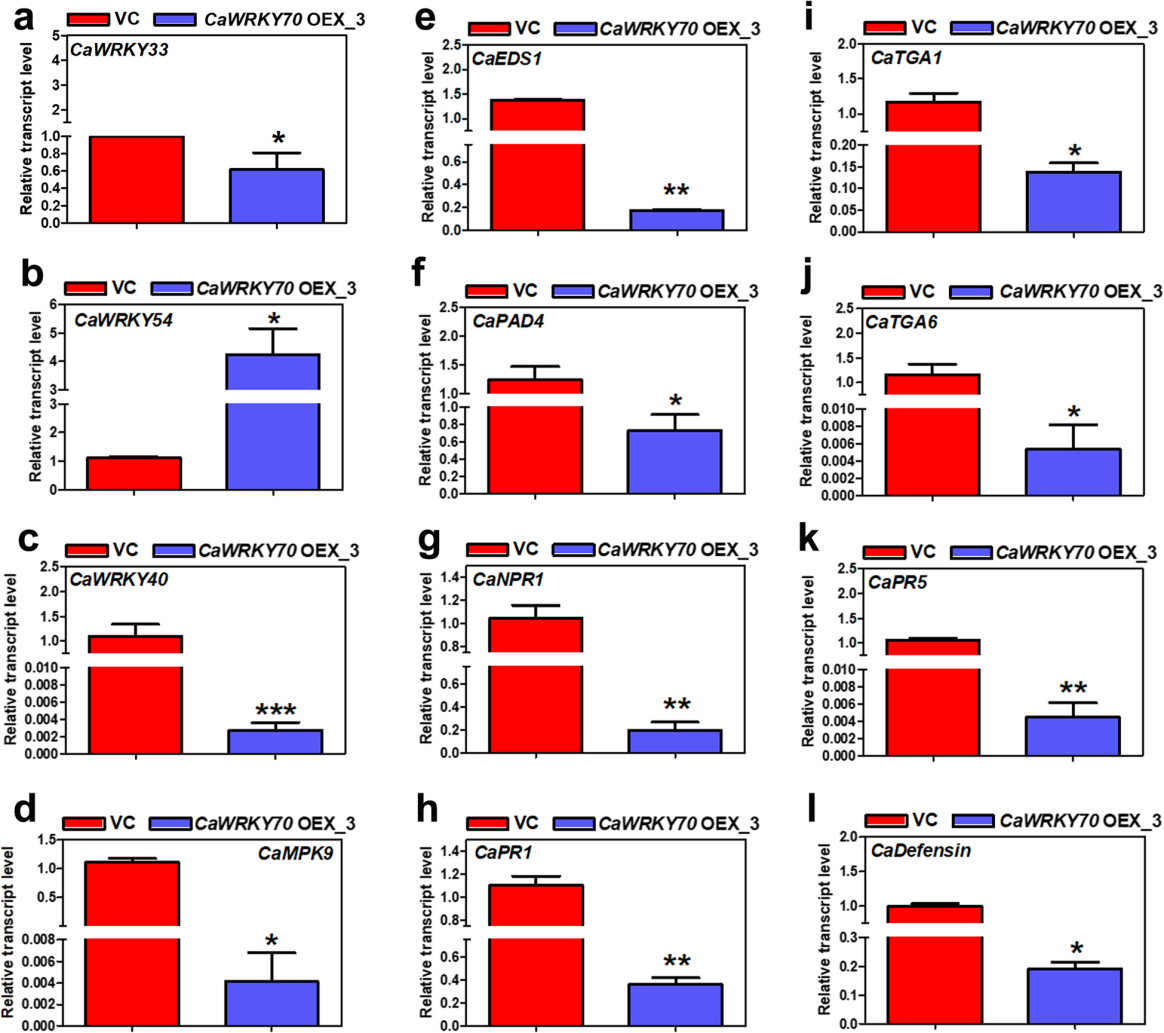
Defense related genes expression in transgenic chickpea on Foc1 stress. **a***CaWRKY33* (XM_004490620.3), (**b**) *CaWRKY54* (based on *Glycine max WRKY54* DQ322698.1), (**c**) *CaWRKY40* (XM_004507020.3), (**d**) *CaMPK9* (XM_004505883.3), (**e**) *CaEDS1* (XM_004506171.2), (**f**) *CaPAD4* (XM_012716750.1), (**g**) *CaNPR1* (XM_012716326.2), (**h**) *CaPR1* (XM_004487759.2), (**i**) *CaTGA1* (XM_027334567.1), (**j**) *CaTGA6* (XM_012715238.2), (**k**) *CaPR5* (AJ487040.1), (**l**) *CaDefensin* (DQ288897.2). Data presented are mean of ±SD (n = 3). Asterisks (*) indicate values are significantly different from control transgenic (VC) plants as measured by Student’s *t* test (*P ≤ 0.05, **P ≤ 0.01 and ***P ≤ 0.001)

We compared the expression of defense responsive transcripts between vector inoculated and *CaWRKY70* overexpressing transgenic chickpea on Foc1 challenge. *CaWRKY33* transcript was remarkably lower at the shoot tissues of overexpressing chickpea than vehicle transgenics (Fig. 5a). WRKY54 and WRKY70 are the defense related transcription factors that negatively regulate osmotic stress tolerance in *Arabidopsis* [41]. At shoot tissues of *CaWRKY70* overexpressing chickpea, *CaWRKY54* mRNA was upregulated after Foc1 treatment (Fig. 5b). *CaWRKY40* expression in *Arabidopsis* enhanced resistance to virulent *Pseudomonas syringae* pv. tomato DC3000 infection [42]. Furthermore, CaMPK9-CaWRKY40 signaling promotes primary defense responses in chickpea against Foc1 [43]. *CaWRKY70* inhibits *CaWRKY40* expression in transgenic chickpea (Fig. 5c). *CaWRKY70* overexpressing chickpea also demonstrates a sharp decrease in *CaMPK9* transcript level (Fig. 5d). EDS1 and PAD4 complex formation was found to be required for pathogen infection induced SA accumulation [44]. However, both *CaEDS1* and *CaPAD4* mRNAs were downregulated in *CaWRKY70* overexpressing chickpea plant type (Fig. 5e, f). Recent finding showed that phosphorylation dependent changes in AtNPR1 promotes its interaction with AtWRKY70 which suppresses *Pathogenesis Related* (*PR*) gene transcription in *Arabidopsis* [45]. Present result suggests that *CaNPR1*, *CaPR1* and *CaPR5* transcript levels were reduced in *CaWRKY70* overexpressing chickpea in comparison to the vehicle treated plants (Fig. 5g, h and k). Among other SA signaling genes, expression of *CaTGA1* and *CaTGA6* mRNAs were downcast in *CaWRKY70* overexpressing chickpea (Fig. 5i, j). Induction of JA-signaling gene *CaDefensin* mRNA is also inhibited at shoot tissues of *CaWRKY70* over-accumulating plants than control vector transformed chickpea (Fig. 5l). Overall, CaWRKY70 negatively regulates induction of defense related genes expression at shoot tissues of transgenic chickpea upon Foc1 infection.

### CaWRKY70 represses *CaWRKY40* promoter activity

**Fig. 6:**
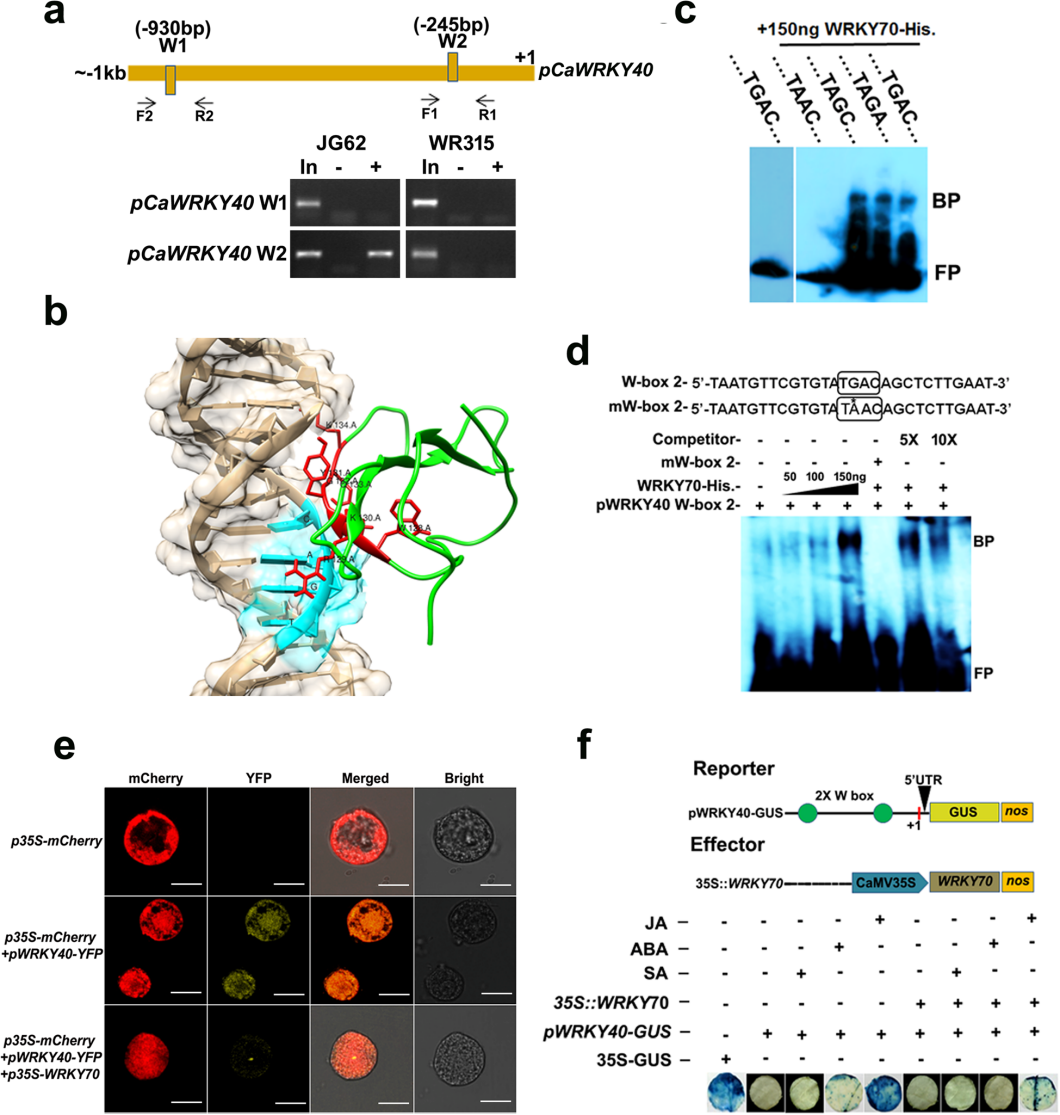
CaWRKY70 suppresses *CaWRKY40* promoter activity in vivo and *in planta*. **a** In vivo Chromatin immunoprecipitation (ChIP) PCR assay shows CaWRKY70 binding to W-box 2 of *CaWRKY40* promoter at shoot tissues of susceptible (JG62) chickpea under Foc1 stress. Diagram shows presence of W-boxes at *CaWRKY40* promoter. (In) denotes input amplified from pre-cleared chromatin samples. Arrow indicates position of the primers. Plus (+) and minus (−) signs indicate anti-CaWRKY70 antibody and pre-immune sera immunoprecipitated chromatins. (+ 1) denotes transcription start site (TSS). **b** In silico molecular docking of CaWRKY70 and W-box 2 containing *CaWRKY40* promoter DNA of cluster 3. **c, d** Electrophoretic mobility shift assay (EMSA) shows in vitro histidine tagged WRKY70 binding at *pWRKY40* W-box 2. Approximately, 200 ng of WRKY70-His protein specifically binds at W-box 2 (− 217 to − 245 bp upstream of TSS). Plus (+) and minus (−) signs indicate presence or absence of specific components. BP indicates bound probe and FP represents free probe. Box indicates W-box 2. Asterisk (*) indicates mutated W-box. The experiment was repeated twice with similar results. **e** CaWRKY70 mediated trans-inhibition of *CaWRKY40* promoter activity. *p35S*:*CaWRKY70* and *pWRKY40*:*YFP* constructs were co-transfected in protoplasts obtained from *Nicotiana tabacum* cv. Xanthi. (Brad) cell suspension culture. mCherry was used as transformation marker. Scale bar = 10 μm. (**f**) CaWRKY70 reduces GUS expression driven by *CaWRKY40* promoter in tobacco leaf discs. Diagram shows constructs used for *Agrobacterium* mediated transient co-infiltration experiment. Plus (+) and minus (−) signs indicate presence or absence of specific vehicles

As described above, *CaWRKY70* overexpressing chickpea significantly lowered the expression of *CaWRKY40* mRNA under Foc1 infection; we got interested to check whether CaWRKY70 plays any direct modulatory role in the suppression of *CaWRKY40* promoter activity. ChIP-PCR assay revealed that CaWRKY70 physically associates with W-box 2 at *CaWRKY40* promoter. CaWRKY70 binding was observed at shoot tissues of susceptible (JG62) chickpea plants under Foc1 stress, whereas resistant chickpea shoot does not exhibit in vivo CaWRKY70 binding (Fig. 6a). Next, we tested this binding through DNA-protein docking experiment. Phyre2 server used WRKY transcription factor 1 (PDB ID: c2aydA) as template structure with 100% confidence and 34% coverage (Additional file 1: Figure S3a-c). Furthermore, qualitative assessment of the predicted model was evaluated by Ramachandran plot analysis using RAMPAGE server. Ramachandran plot analyses revealed that in CaWRKY70 model 94.7% residues are in favoured region, 5.3% residues are in the allowed region and none of the residues in outlier location (Additional file 1: Figure S3d-e). For understanding the molecular mechanism of its interaction with W-Box DNA, an in silico DNA-protein docking was carried out with HADDOCK (Fig. 6b). HADDOCK web server clustered 174 structures in 12 clusters, representing 87.0% of the HADDOCK generated water-refined models. Clusters were ranked according to HADDOCK score and Z-score for plotting DNA-protein interaction. Top five best scoring clusters are provided in Additional file 2: Table S1. HADDOCK score is calculated as the weighted sum of van der Waals, electrostatic, desolvation and restraint violation energies whereas, the Z-score indicates how many standard deviations from the average of these clusters is in terms of score. For predicted protein model, HADDOCK score v/s i-RMSD (interface-RMSD) plot was created. i-RMSD was calculated based on the backbone (CA, C, N, O, P) atoms of all residues involved in intermolecular contact using 10 Å cut-off. l-RMSD (ligand-RMSD) was also calculated on the backbone atoms of all (*N* > 1) molecules (Additional file 2: Table S1, Fig. 6b). DNA-protein interaction was further confirmed by in vitro EMSA. Result indicates in vitro CaWRKY70 binding to wild-type (TGAC), and mutated W-boxes (TAGC and TAGA) (Fig. 6c). Approximately, 150 ng of purified histidine tagged CaWRKY70 exhibits strong DNA binding to W-box 2 at *CaWRKY40* promoter. However, such binding was completely inhibited upon mutating G nucleotide of TGAC i.e., TAAC. In vitro DNA binding of CaWRKY70 was outcompeted using 5 and 10 M excess cold probes, respectively (Fig. 6d). Effect of CaWRKY70 binding at W-box 2 of *CaWRKY40* promoter was tested by transient co-infiltration experiments using *Nicotiana xanthi* protoplasts and *Nicotiana tabacum* leaf discs, respectively. Protoplast co-transfection experiment showed that CaWRKY70 effectively inhibits *CaWRKY40* promoter mediated expression of YFP in *N. xanthi* protoplast, which suggests CaWRKY70 mediated negative regulation of *CaWRKY40* promoter activity in vivo (Fig. 6e). *Agrobacterium*-mediated transient co-infiltration of *p35S:CaWRKY70* (effector construct) and *pCaWRKY40:GUS* (reporter construct) in tobacco leaf discs demonstrates reduction in *CaWRKY40* promoter driven GUS expression (Fig. 6f). Quantitative data also revealed ~ 2.5-fold reduction in the histochemical GUS staining upon constitutive induction of CaWRKY70 (Additional file 1: Figure S4). Therefore, CaWRKY70 binds to and represses *CaWRKY40* promoter activity.

### CaWRKY40 positively regulates *CaMPK9* promoter activity

**Fig. 7:**
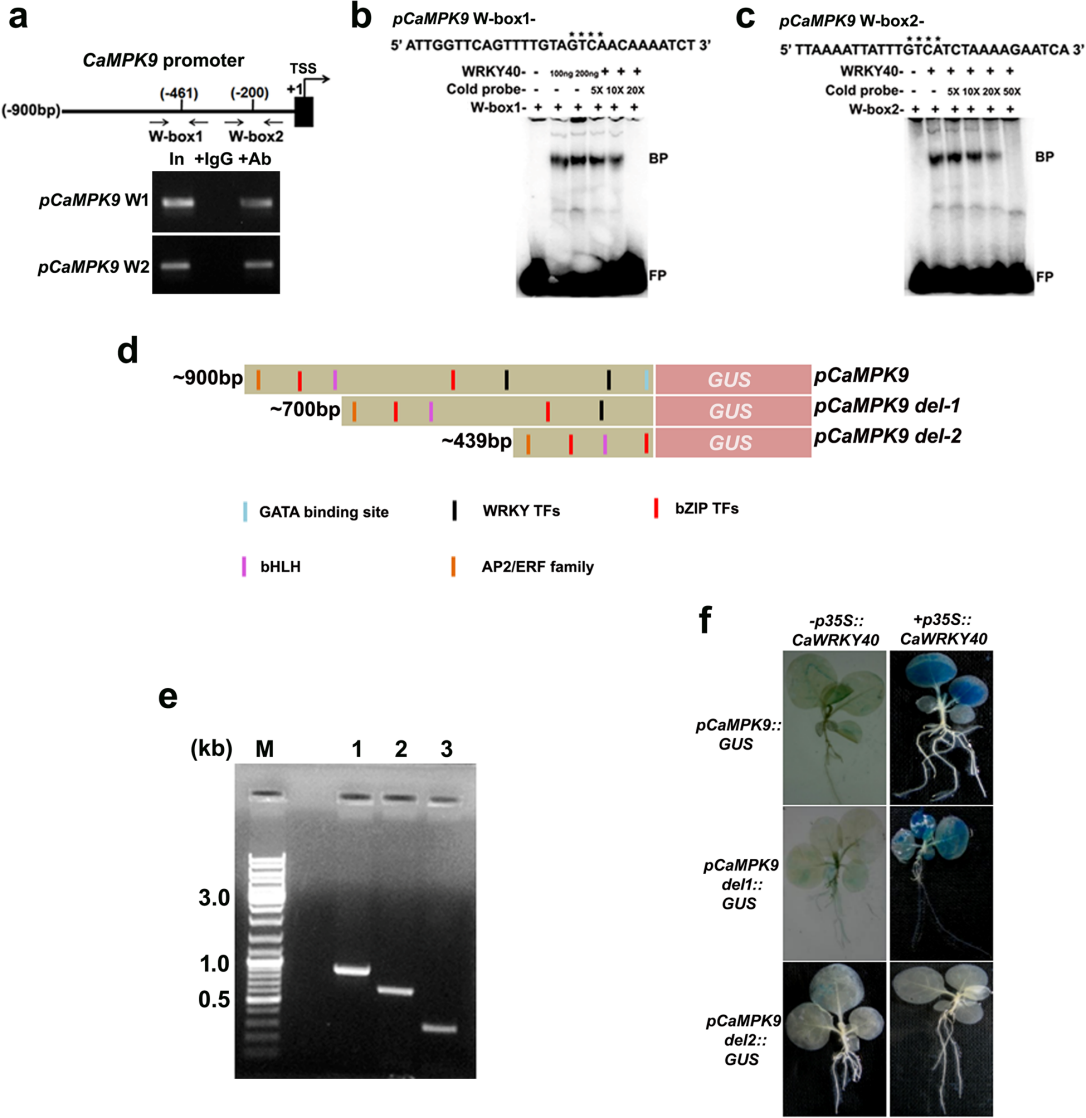
CaWRKY40 upregulates the activity of *CaMPK9* promoter *in planta*. **a** In vivo binding of CaWRKY40 to *CaMPK9* promoter at W-boxes. Schematic represents *CaMPK9* promoter and the W-boxes relative to transcription start site (+ 1). Arrows indicate the sites for primer binding. *In planta* immunoprecipitation of WRKY40-chromatin complex from susceptible and resistant chickpea shoots with anti-CaWRKY40 antibodies. Bound chromatins were eluted and used for PCR reactions. Sheared pre-cleared chromatin served as input control. Rabbit IgG was used as negative control for ChIP PCR. **b, c** In vitro binding of recombinant WRKY40 to W-boxes at *CaMPK9* promoter. Approximately, 200 ng of His-WRKY40 protein was added to W-box containing labeled *CaMPK9* promoter fragment in an independent EMSA reaction. BP and FP indicate bound and free probes, respectively. Plus (+) and minus (−) signs denote presence or absence of individual elements. Asterisks highlight the W-boxes. Experiments were repeated twice with similar results. **d** Diagrams show 3’deletion fragments of *CaMPK9* promoter and presence of the cis regulatory elements. **e** Genomic amplification of *CaMPK9* promoter derivatives in transgenic tobacco by PCR. **f** Transactivation of *pCaMPK9:GUS* in transgenic *N. tabacum* seedlings upon transient expression of *p35S:CaWRKY40*

Recent report shows that higher activation of CaMPK9 in resistant chickpea phosphorylates CaWRKY40 under Foc1 stress [43]. We further anticipated that phosphorylated CaWRKY40 positively regulates *CaMPK9* expression via feed-back mechanism in resistant chickpea on Foc1 challenge. Here, increased expression of *CaMPK9* transcript in resistant chickpea also suggests its positive regulatory role in the defense activation against Foc1. Transcript level was found to be ~ 3.0-fold higher in resistant chickpea plants over control treatment. By contrast, susceptible chickpea plants show sharp downregulation of *CaMPK9* transcript upon Foc1 challenge (Additional file 1: Figure S5). ChIP-PCR data supports in vivo association of CaWRKY40 with W-boxes at *CaMPK9* promoter in resistant genotype plants upon exposed to Foc1 (Fig. 7a). EMSA was used to establish in vitro binding of recombinant WRKY40 protein at *CaMPK9* promoter DNA. Binding reactions were performed using 200 ng purified 6 × histidine-tagged WRKY40 protein. Result shows the formation of sharp bound complexes after incubation of recombinant WRKY40 protein and labelled W-boxes. The complexes were competed out using 20 (for W-box 1) or 50 (for W-box 2) molar excess cold competitors (Fig. 7b, c). To ascertain the specific role of CaWRKY40 in *CaMPK9* promoter activation, W-box-specific deletion constructs were generated and stably transformed into tobacco genome by *Agrobacterium* mediated gene transfer method (Fig. 7d). Integration of the *CaMPK9* promoter fragments within tobacco genome was further confirmed by genomic PCR. Result shows sharp amplification of *CaMPK9* promoter deletion derivatives (Fig. 7e). *CaMPK9* promoter activity was further monitored in the presence or absence of specific effector constructs that constitutively express CaWRKY40. Reduction in the GUS activity was highest when both W-boxes were deleted (Fig. 7f). However, deletion of a single W-box results in mild reduction of the GUS activity in effector construct-infiltrated setup. These results suggest that CaWRKY40 binds at *CaMPK9* promoter via both W-box 1 and W-box 2, which in turn positively modulates *CaMPK9* expression.

### Physical interaction between CaWRKY70 and CC-NB-ARC-LRR protein suppresses cell death in chickpea

**Fig. 8:**
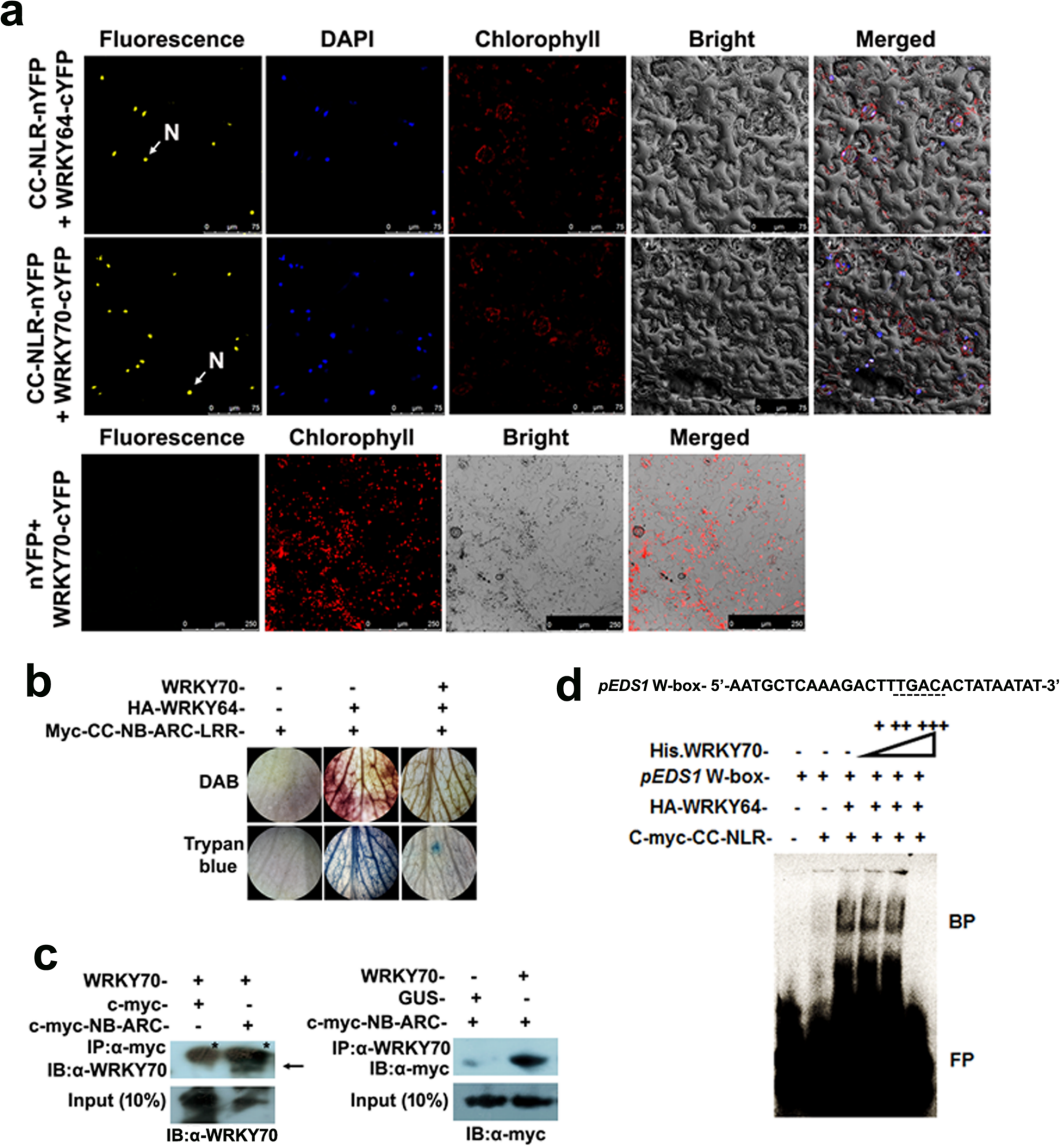
CaWRKY70 attenuates R-protein signaling in chickpea. **a** BiFC assay for physical interaction between CC-NLR-nYFP and CaWRKY70-cYFP. (nYFP + CaWRKY70-cYFP) and (CC-NLR-nYFP + CaWRKY64-cYFP) interactions were used as negative and positive controls, respectively. N denotes nucleus. **b** H_2_O_2_ accumulation and cell death in chickpea leaves. Myc-CC-NLR, HA-WRKY64 and CaWRKY70 were transiently co-expressed in chickpea leaves by *Agrobacterium*. Infiltrated chickpea leaves were subjected to DAB and trypan blue staining. **c** Co-immunoprecipitation of c-myc-NB-ARC and CaWRKY70 after *Agrobacterium* mediated transient expression in *N. benthamiana* leaves. Arrow indicates the immunoprecipitated protein band of CaWRKY70. Asterisks show non-specific bindings. **d** His.-WRKY70 binding at W-box of chickpea *EDS1* promoter by EMSA. Dotted lines represent W-box. BP and FP indicate bound and free probes, respectively. In **b, c** and **d**, plus (+) and minus (−) signs indicate presence or absence of specific components

The present group has recently established that physical interaction between RPP2-like CC-NB-ARC-LRR (CC-NLR) protein and CaWRKY64 triggers *in planta* ectopic cell death [30]. To further investigate whether CaWRKY70 similarly influences cell death signaling by CC-NLR protein, we tested their *in planta* interaction through bimolecular fluorescence complementation (BiFC) assay using *Nicotiana benthamiana* leaves. Results demonstrate that CaWRKY64 and CaWRKY70 tagged to C-terminus of YFP (cYFP) interact with full-length CC-NLR protein fused to N-terminus of YFP (nYFP). Result shows that reconstitution of YFP signal was observed in the nucleus (Fig. 8a). However, no such interaction was detected with control vector i.e., WRKY70-cYFP+nYFP. After testing their potential interaction between CaWRKY70 and CC-NLR protein, we were curious to check the effect of CaWRKY70 interaction on CaWRKY64 and CC-NLR protein mediated cell death phenomenon. Thus, we co-expressed HA tagged CaWRKY64, CaWRKY70 and myc tagged CC-NLR protein in chickpea leaves by *Agrobacterium*. The infiltrated leaves were further subjected to histochemical DAB and trypan blue staining. DAB staining shows that co-expression of epitope tagged CaWRKY64 and CC-NLR protein results in high levels of H_2_O_2_ accumulation in infiltrated chickpea leaves, which is suppressed upon CaWRKY70 expression (Fig. 8b). Similarly, trypan blue staining also depicted that CaWRKY70 effectively inhibits the retention of blue colouration and cell death in chickpea leaves when myc tagged CC-NLR protein and HA tagged CaWRKY64 were co-expressed (Fig. 8b). Next, we performed co-immunoprecipitation (Co-IP) to show physical interaction between myc epitope tagged NB-ARC domain of CC-NLR protein and CaWRKY70. Proteins were transiently co-expressed in *N. benthamiana* leaves by *Agrobacterium*. Reciprocal Co-IP analyses detected that CaWRKY70 and myc-NB-ARC proteins were co-precipitated by anti-myc and anti-WRKY70 antibodies, respectively. Immunoblotting was performed with anti-WRKY70 and anti-myc antibodies. Similarly, input samples show the presence of both CaWRKY70 and myc-CC-NLR proteins after probed with anti-WRKY70 and anti-myc antibodies (Fig. 8c). Effect of CaWRKY70 interaction on CC-NLR and CaWRKY64 mediated DNA binding has been further tested by in vitro EMSA experiment. Our group has previously shown that CC-NLR protein stimulates in vitro DNA binding of epitope tagged CaWRKY64 protein at *CaEDS1* promoter [30]. This binding was found to be reduced by the addition of an increasing amount of recombinant his-tagged WRKY70 protein (Fig. 8d). Together, we establish that physical interaction between CaWRKY70 and CC-NLR protein negatively regulates cell death signaling in chickpea.

## Discussion

WRKY70 transcription factor is well characterized as an important transcriptional modulator of SA mediated signal transduction pathways in *Arabidopsis* and wheat [39, 46]. *AtWRKY70* overexpressing *Arabidopsis* plants demonstrated enhanced resistance to biotrophic pathogens *Pseudomonas syringae* and *Erysiphe chichoracearum* [39]. However, the plants showed hyper-susceptibility to necrotrophic fungus *Alternaria brassicicola* [9]. Contrastingly, *Atwrky70* mutants displayed susceptibility towards *B. cinerea* infection [47]. In wheat, *TaWRKY70* positively regulates defense against stripe rust pathogen *Puccinia striiformis* f. sp. *tritici* [46]. Both *Arabidopsis* and chickpea WRKY70 are group III members have been found to be positively involved in defense reaction to bacterial pathogen *P. syringae* [3, 42, 48]. Weak interaction of AtWRKY46 to AtWRKY70 and AtWRKY53 positively regulate the basal defense responses in *Arabidopsis* [48]. AtWRKY46, AtWRKY70, and AtWRKY53 notably suppress JA-induced defense genes expression. Thus, involvement of WRKY70 protein in plant defense events is dynamic and host-pathogen specific. Despite its contribution in plant defense related functions, present group has already established its direct negative regulatory effects on abiotic stress responses in chickpea [49]. Here, we decipher immune suppressive functions of CaWRKY70 in chickpea shoots upon Foc1 infection.

SA response is common for induction of several *WRKY* genes involved in distinct stages of the SAR activation in plants [50, 51]. SA treatment appears to be a positive inducer of SAR development and *CaWRKY70* expression in chickpea. Its mRNA level increases approximately 5-fold after 6 h of SA application over control treatment in susceptible plant compared to the resistant one (Fig. 1). Likewise, *AtWRKY70* expression was also found to be induced almost 30-fold at 2 h post- SA treatment [9]. SA signaling invokes *AtWRKY70* expression in young and senescing leaves. Our result shows that *CaWRKY70* expression was highest at shoot tissues of chickpea (Fig. 1). Constitutive expression of bacterial salicylate hydroxylase *NahG* removes free SA and the subtle induction of *AtWRKY70* transcript [52]. *Arabidopsis* mutants *eds1*, *pad4* and *npr1* compromised in SA signaling demonstrate reduced level of *AtWRKY70* transcript, whereas mutant plants *edr1*, *cpr5* and *acd11* exhibited SA hyperaccumulation and subsequent induction of *AtWRKY70* [53, 54]. SA and JA are two antagonistic signaling molecules that influence plant defense [55]. However, JA treatment was found to be ineffective for *CaWRKY70* transcript induction. In most cases, WRKY70 promotes SA-responsive genes expression and inhibits subset of JA-responsive genes [9]. Enhanced expression of *AtWRKY70* in *coi1* mutant suggests that JA-responsive factor represses *AtWRKY70* expression depending on endogenous JA levels [9]. Although, CaWRKY70 suppresses the expression of SA biosynthesis and signaling genes at shoot tissues of transgenic chickpea upon Foc1 infection (Figs. 4 and 5). *ICS1* and *PAL* expression positively influences activation of SA signaling pathways and phenyl propanoid biosynthesis pathways leading to the production of anti-microbial secondary metabolites that protects chickpea and tomato plants from nematode penetration and *Fusarium oxysporum* infection, respectively [56, 57]. Our previous transcriptomic and metabolite analyses also revealed induction of *CaICS1* and *CaPAL* transcripts and associated SA accumulation in resistant (WR315) chickpea after Foc1 inoculation [38]. By contrast, *CaICS1*, *CaPAL* expression and SA concentrations were significantly depleted at shoot tissues of *CaWRKY70* overexpressing chickpea under Foc1 stress (Fig. 4). EDS1 and PAD4 are two such important regulatory components of SA biosynthesis in plants upon pathogen stress [44, 58]. In chickpea, we previously observed constant induction of *CaEDS1* and *CaPAD4* transcripts at both shoot and root tissues of resistant genotypic plant under Foc1 infected condition [38]. However, *CaEDS1* and *CaPAD4* transcripts were downregulated at *CaWRKY70* overexpressing chickpea shoot tissues in response to Foc1 infection (Fig. 5). This may strengthen negative regulatory role of CaWRKY70 in systemic defense reactions. SA signaling in plants depends on the activation of TGA transcription factors and NPR1. These two transcriptional modulators synergistically control expression of the two critical SA marker genes i.e., *PR1* and *PR5*. The effective induction of *CaTGA1* and *CaTGA6* mRNAs were observed at shoot tissues of Foc1 infected resistant chickpea, whereas susceptible plants were unable to stimulate such mRNA accumulation [38]. CaWRKY70 expresses at shoot tissues of susceptible chickpea after Foc1 inoculation and its overexpression in resistant chickpea plants markedly reduces *CaTGA1* and *CaTGA6* transcripts accumulation (Figs. 1 and 5), which indicates to the impairment of conserved SA signaling in susceptible chickpea. *CaNPR1*, *CaPR1* and *CaPR5* transcripts follow the same pattern of Foc1 induced downregulation at shoot tissues of *CaWRKY70* overexpressing chickpea than control transgenics (Fig. 5). Importantly, CaWRKY70 represses the expression of SA and JA-marker genes i.e., *CaPR1*, *CaPR5* and *CaDefensin* that promotes susceptibility in transgenic chickpea (Fig. 5). SA-mediated repression of JA-responsive gene expression is governed by cytosolic NPR1 [59]. WRKY70 controls JA-repressors based on cytosolic modification of NPR1 protein [59]. CaWRKY70-mediated suppression of *CaNPR1* might play negative role in SA-responsive *CaPR1*, *CaPR5* and JA-induced *CaDefensin* gene expression in chickpea (Fig. 5). *PDF1.2* transcript in *Atwrky70* mutant was found to be low which enhanced upon *B. cinerea* infection. However, in *CaWRKY70* overexpressing chickpea, *CaDefensin* expression has been downregulated under Foc1 infection [47]. Low levels of SA promote *PR* genes expression in chickpea, whereas high concentrations inhibit both SA and JA signaling pathways. Thus, CaWRKY70 acts as an integrator of SA and JA responses in the regulation of chickpea defense responses to Foc1. Mutual antagonism and interaction between SA and JA pathways are common regulatory steps for *CaWRKY70* expression and its activation that governs wilt-disease resistance phenomenon in chickpea. Our previous study revealed that differential SAR induction in resistant and susceptible chickpea plants are analogous to the SA dependent gene expression and here, we convey mechanism of its attenuation. Hence, our present study explains the complexity of SA biosynthesis, signaling and its feed-back inhibition by CaWRKY70 that control Foc1 resistance/susceptibility in two contrasting chickpea accessions, respectively.

Regulatory sequences and DNA-binding activity of WRKY family members remarkably govern various cellular and stress responsive phenotypes in plants [60]. DNA binding role of CaWRKY70 is not an unusual phenomenon since, it is a nuclear localized protein (Additional file 1: Figure S1). In Foc1 infected chickpea, CaWRKY70 inhibits the expression of *CaWRKY40* signaling genes i.e., *CaWRKY33* and *CaMPK9* (Figs. 5 and 7). Recent finding suggests CaMPK9 interaction and phosphorylation provide stability to CaWRKY40 protein in chickpea upon Foc1 infection [43]. CaWRKY40 mediated upregulation of *CaMPK9* expression was suppressed in *CaWRKY70* overexpressing chickpea. Shared transcriptional regulation of *At*WRKY18, *At*WRKY40 and *At*WRKY60 adjusts abscisic acid (ABA) signaling mediated abiotic stress responses in plants [61]. We found that CaWRKY70 binds at *CaWRKY40* cis-elements and represses its activity (Fig. 6). It is interesting to note that CaWRKY40 positively regulates the *CaMPK9* promoter activation which has been established by both in vivo and *in planta* experiments (Fig. 7). *CaMPK9* upstream elements deletion study also revealed its role in the modulation of promoter activity. CaWRKY70 activated transcription of *CaWRKY54* gene in transgenic chickpea (Fig. 5). These two transcription factors co-ordinately function as negative regulators of leaf senescence, stomatal closure, and osmotic stress tolerances in *Arabidopsis* [41, 62]. Although, our study demonstrated that CaWRKY70 and CaWRKY54 cooperatively contribute to Foc1 susceptibility in chickpea. CaWRKY70 mediated promoter modulation suggests bidirectional transcriptional regulation. Therefore, CaWRKY70 mediated inhibition of appropriate immune signaling in chickpea accomplishes through its direct and indirect negative regulatory influence on defense genes expression under Foc1 stress condition.

Transcriptional responses behind SAR activation by WRKY proteins were previously established in *Arabidopsis* [63, 64]. However, the mechanism of its repression is not known. Present study shows that deactivation of SAR in pathogen-free systemic tissues of chickpea is mediated by CaWRKY70 upon Foc1 infection. Repressor activity of AtWRKY70 on *SARD1* gene expression regulates the balance between growth and defense in *Arabidopsis* [19, 20]. Such regulatory functions are yet to be demonstrated in chickpea. CaWRKY70 is transcribed and translated at susceptible chickpea shoots upon Foc1 infection (Fig. 1). Since, SAR activation was prominent at shoot tissues of resistant chickpea [38], we monitored *CaWRKY70* over-expression effect in this background. The overall suppression of SA signal transduction network confers Foc1 susceptibility in *CaWRKY70* overexpressing chickpea (Figs. 4 and 5). Although, it is tempting to monitor *CaWRKY70* knock-down effect in susceptible chickpea background as future attempt.

**Fig. 9:**
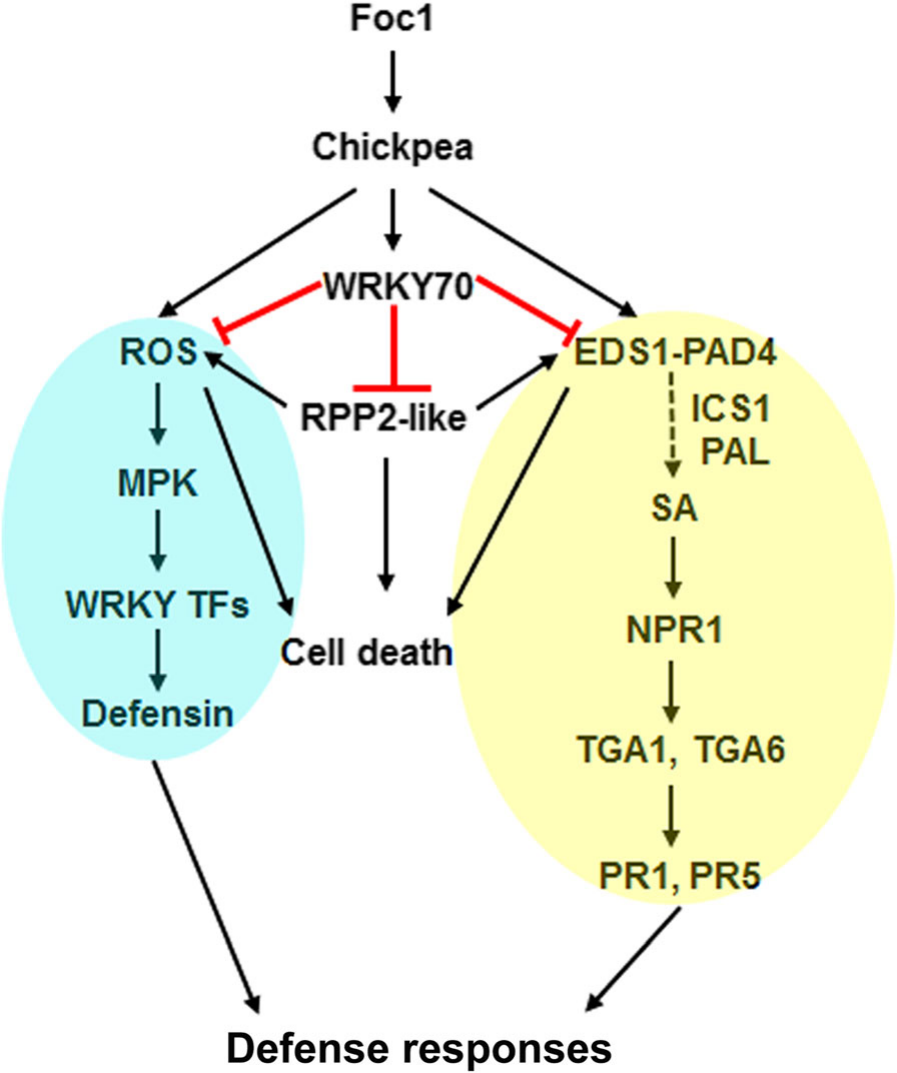
Proposed model for CaWRKY70 mediated suppression of innate immune signaling in chickpea under Foc1 stress. Model depicting negative regulatory role of CaWRKY70 on ROS signaling, MPK-WRKY pathway, SA signaling and RPP2 mediated cell death

SA has immense roles in plant immunity, including resistance gene signaling. EDS1 is a well-known genetic regulator of SA production, resistance gene functioning and cell-death [65]. RPP2-like CC-NB-ARC-LRR protein and CaWRKY64 mount EDS1 dependent ectopic defense activation and cell-death in chickpea [30]. EDS1 mostly confers resistance as part of the TIR-NB-LRR signaling [66]. Based on our protein-protein interaction studies, we establish that physical interaction between CaWRKY70 and chickpea RPP2-like CC-NLR protein effectively suppresses ROS accumulation and cell-death induction *in planta* (Fig. 8). RPP4-mediated resistance response against *Hyaloperonospora parasitica* Emoy2 was partially reduced in *Atwrky70* mutants [32]. On the other hand, RPP7-triggered defense reaction was not affected in this mutant background. Thus, R-protein mediated defense response pathway is positively correlated with AtWRKY70 functioning in *Arabidopsis*, whereas it is inhibited by CaWRKY70 in chickpea might contradict WRKY70 dependent R-protein signaling in general. CaWRKY70 mediated inhibition of ROS accumulation in transgenic chickpea root, stem and leaves also support our interpretation (Fig. 3). Although, reduction in oxidative bursts signaling and SA production does not fully correlate with the increased ion-leakage and fungal biomass accumulation in *CaWRKY70* overexpressing chickpea root than control transgenics (Figs. 2 and 3). We reasoned that electrolyte leakage is associated with membrane damage due to higher colonization of Foc1 biomass, whereas ROS accumulation induced cell death is a defense phenomenon that inhibits *in planta* Foc1 growth. Importantly, less cell death promotes higher Foc1 colonization in *CaWRKY70* overexpressing chickpea roots. On the other hand, complete inhibition of EDS1 signaling not only affects SA induction, but also cell-death promotion (Figs. 5 and 8). In summary, CaWRKY70 functions as nodal suppressor that includes fundamental defense regulators like, SA response and EDS1 into R-protein mediated signal transduction pathways highlighted in chickpea upon Foc1 stress (Fig. 9).

## Conclusions

Finally, this study provides information which may fill the gaps between already available knowledge about CaWRKY70 mediated transcriptional control of downstream defense signaling pathways. Promoter occupancy and protein-protein interaction play crucial roles for suppression of chickpea defense to Foc1. Interpretation of our findings may be translated and recapitulated for serial examination of multiple layers of defense signaling in chickpea. Repressor role of CaWRKY70 in modulating ROS homeostasis, SA biosynthesis and signaling is an interesting finding. Interconnection between several signaling cues in turn confer resistance against Foc1 in definite ways depending on time point of infection, duration, and severity. Notably, spatiotemporal expression patterns of *CaWRKY70* mediated immune signaling elements renovates our apprehension. Thus, present study is useful to develop strategies for protecting chickpea from *Fusarium* wilt disease.

## Methods

### Plant materials and growth conditions

Experiments were performed using two different genotypes of chickpea (*Cicer arietinum* L.) i.e., JG62 (wilt susceptible) and WR315 (wilt resistant) obtained from Dr. Suresh C. Pande, ICRISAT (International Crops Research Institute for Semi-Arid Tropics), Patancheru, Andhra Pradesh, India. Surface sterilized seeds of both genotypes were sown in the pots containing autoclaved mixture of soil-rite and soil under natural greenhouse conditions at 22 to 25 °C temperature, 70% relative humidity, 100 μ mol m^− 2^ s^− 1^ light intensity and 16 h photoperiod. Pots were watered from bottom at every 2 days and supplemented with half strength Hoagland’s medium (TS1094, Hi-media Laboratories, Mumbai, India). *Nicotiana tabacum* L. cv. Samsun NN and *N. benthamiana* seeds were gifted by Dr. Nrisingha Dey, Institute of Life science, Bhubaneshwar, India. Seeds were surface sterilized and grown aseptically on MS medium at 24 °C temperature and 60% relative humidity with light intensity of 100 μ mol m^− 2^ s^− 1^ under 16 h photoperiod.

### Fungal inoculation

*F. oxysporum* f. sp*. ciceri* Race1 (Foc1) fungal strain was obtained from Dr. Suresh C. Pande, International Crops Research Institute for the Semi-Arid Tropics (ICRISAT), Patancheru, Andhra Pradesh, India. Sixteen-days-old chickpea plants were inoculated with Foc1 using sick-soil method according to the previously described method [34, 43].

### Hormone treatment

For inducer treatments, 2 mM salicylic acid (SA, Hi-media), 100 μM ABA (Abscisic acid, Hi-media) and 50 μM JA (Jasmonic acid, Hi-media) was sprayed on greenhouse gown sixteen-days-old chickpea plants of both susceptible and resistant accessions. Leaf tissues were collected at 6 h of treatment for RNA isolation.

### RNA isolation and quantitative real-time PCR (qRT-PCR) analyses

Shoot tissues of sixteen-days-old susceptible and resistant chickpea plants were collected and frozen in liquid nitrogen. Total cellular RNA was extracted from frozen sample using TRIZOL reagent (Himedia). First strand cDNA synthesis was carried out from 5 μg total RNA using First Strand cDNA synthesis Kit (Thermo Scientific, USA) following manufacturer’s guideline. qRT-PCR was performed using Bio-Rad iCycler (Bio Rad iQ5) with SyBr green (Bio Rad). The reaction mix containing SyBr green qPCR Super Mix (2×) (Bio Rad), 25 ng cDNA, and 0.3 μM of forward and reverse primers (Additional file 3: Table S2). Chickpea *Glyceraldehyde-3-phosphate dehydrogenase* (*CaGAPDH*) expression was used as internal control. Normalized fold change levels for all the genes were calculated using the 2 ^−ΔΔ(Ct)^ method [67].

### Subcellular localization

For subcellular localization study, YFP gene was cloned in *BamH*I/ *Sac*I site of pBI121binary plant transformation vector. Full-length *CaWRKY70* gene was PCR amplified and fused in frame to N terminal part of the yellow fluorescent protein (*YFP*) gene in pBI121 vector for preparation of the *35S:WRKY70-YFP* construct. The vectors were mobilized into the competent *Agrobacterium tumefaciens* strain GV3101. *35S:WRKY70-YFP* and control *35S:YFP* vectors were transiently transformed into onion epidermal cells by *Agrobacterium*. At 48 h post-agroinfiltration, epidermal cells were observed under a confocal microscope (Leica TCS SP2 AOBS system) to monitor localization patterns of fusion proteins under excitation and emission at 514 nm and 527 nm, respectively.

### Molecular cloning

Full-length coding sequence of *CaWRKY70* (GenBank Accession No. XM_004502763.3) was amplified from chickpea cDNA pool by reverse-transcriptase polymerase chain reaction (RT-PCR) using gene specific primers (Additional file 3: Table S2). Purified PCR amplicons were restriction digested with *BamH*I/ *Xho*I (Roche, Mannheim, Germany) and cloned in modified pBI221 vector containing short multi-cloning sites (MCS) region by replacing *GUS* gene. Cassette was gel excised after treatment with *Hind*III/ *EcoR*I (Roche, Mannheim, Germany) and cloned in MCS of binary plant expression vector pCAMBIA2301. Cloning was checked by restriction digestion, which is followed by sequencing of the full-length gene. Binary plant transformation vector containing *CaWRKY70* gene was mobilized to *Agrobacterium tumefaciens* strain AGL-1. Empty pCAMBIA2301 vector was also transformed into *Agrobacterium* strain AGL-1 was used as vector control.

### Chickpea transformation

*Agrobacterium*-mediated chickpea transformation was carried out as described by [68] with a modified rooting protocol [69]. Briefly, chickpea transformation was carried out with single cotyledon and half-embryo explant followed by infection with *Agrobacterium* strain AGL-1 harbouring empty pCAMBIA2301 vector and modified vector carrying *CaWRKY70* gene. Multiple shoots were regenerated from the explants and elongated with 0.25 mg/ l IAA (Indole-3-acetic acid) for 10 days. The elongated shoots were transferred to rooting medium (1/2 MS salts, B5 vitamins, 1 mg/ l IBA and 20 g/ l sucrose) [70]. Finally, rooted plantlets were properly hardened, transferred to glasshouse and established in the pots. Empty pCAMBIA2301 vector generated plants were used as control transgenics whereas, *CaWRKY70* gene carrying modified vector transformed plants were considered as overexpressing chickpea.

### Disease intensity index

Disease intensity index was determined on control transgenic and *CaWRKY70* overexpressing chickpea based on the development of foliar symptoms at 0, 3, 7 and 12 dpi, respectively. Incidence of foliar symptoms (I) was set at 0 to 1 scale and disease severity (S) rated on a 0 to 4 scale (0 - no wilting; 1 - less wilting; 2 - partial wilting; 3 - wilting; 4 - severe wilting). Disease intensity index (DII) was calculated as DII = (I × S)/4 [71].

### Incidence of dead plants

Development of Foc1 infection on control transgenic and *CaWRKY70* overexpressing chickpea in the greenhouse experiment was recorded as incidence of dead plants at 0, 3, 7 and 12 dpi. The percentage of incidence of dead plants from transgenic chickpea was measured using the formula i.e., percentage of incidence of dead plants = (total number of infected plants/ total number of plants assessed) × 100 [72].

### Chlorophyll estimation

For chlorophyll extraction, one gram of control transgenic and *CaWRKY70* overexpressing chickpea leaf samples were crushed with 2 ml dimethyl sulfoxide (DMSO): acetone (1:1vol/ vol) mix. The samples were then kept in a refrigerator at 4 °C for 4 h. The samples were then centrifuged at 500 rpm for 5 min. Following this, supernatant was transferred to fresh 2 ml eppendorf tubes. The colour absorbance (A) of extracts was determined using Shimadzu UV 1800 spectrophotometer (Shimadzu Corporation, Kyoto, Japan) at 645 and 663 nm wavelength against the blank solvent containing 80% acetone. Chlorophyll A and B content was estimated based on [73].

### DAB staining

H_2_O_2_ accumulation in treated *CaWRKY70* overexpressing chickpea root, stem and leaves were visualized by 3, 3′-diaminobenzidine (DAB) staining, according to the method of [74]. The plant tissues were immersed in 1 mg/ ml DAB (3, 3′-diaminobenzidine) solution (pH − 3.8) and vacuum infiltrated for 2 h followed by incubation of 8 h at room temperature. Chlorophyll was removed by incubating in 96% ethanol for overnight and photographed with a digital camera. DAB stained samples were oven dried for 24 h and crushed with sterile double distilled water to measure their intensities by spectrophotometer at 500 nm wavelength and water as blank.

### Trypan blue staining

For trypan blue staining, lactophenol-trypan blue solution was prepared by mixing 10 ml lactic acid, 10 ml glycerol, 10 ml distilled water, 10 g of phenol and 10 mg of trypan blue. H_2_O_2_ treated and Foc1 infected control and transgenic chickpea root, stem and leaves were subjected to trypan blue staining. Plant tissues soaked in trypan blue solution were warmed in a boiling water bath for 1 min and cleared with saturated chloral hydrate solution (2.5 g chloral hydrate dissolved in 1 ml distilled water) for 10 min. Decolorized plant samples were photographed. Trypan blue stained samples were dried and extracted with sterile double distilled water. The crude extracts were quantified using spectrophotometer at 500 nm wavelength against water as blank.

### Bacterial expression and protein purification

PCR amplified full-length *CaWRKY40* and *CaWRKY70*genes were inserted into *EcoR*I/ *Xho*I site of pET28^a(+)^ (Novagen, Germany) and transformed into *Escherichia coli* BL21 (DE3) cells. Recombinant WRKY40 protein purification was carried out as previously described by [42]. Histidine tagged WRKY70 protein purification was performed according to previously described method by [49]. Protein induction was carried out with 1 mm isopropyl thio-β-D-galactoside (IPTG) at 37 °C for 1 h with vigorous shaking (160 rpm) and cells were harvested by centrifugation at 8000 rpm 4 °C for 5 min. Hexa-histidine tagged WRKY70 protein was purified from cell lysate by Ni-NTA affinity chromatography (Qiagen).

### Antibody production

Anti-WRKY70 polyclonal antibodies were raised in rabbits. Two rabbits were immunized for antibody production. Rabbits were injected on 5 occasions with recombinant hexa-histidine tagged WRKY70 protein of 1.5 mg concentrations at every 3-week intervals. Serum obtained from each immunized rabbit was tested 2-weeks after each injection. Pre-immune serum was collected from each animal. Approximately, 20–30 ml serum/ rabbit was obtained. The serum was affinity purified. Antibodies were used at a final dilution of 1:10,000 for immunoblotting experiments.

### Protein extraction and immunoblotting

Total soluble protein extraction from sixteen-days-old control and Foc1 inoculated susceptible and resistant chickpea shoots were performed using an ice-cold protein extraction buffer (50 mM Tris−HCl pH 7.5, 100 mM NaCl, 1 mM DTT, 0.5% Triton X-100, 0.1% SDS and 10% glycerol) followed by the addition of protease inhibitor cocktail (ETDA-free, Roche). Protein concentration was measured by Bradford assay [75]. Approximately, 20 μg of total soluble protein was separated by 10% sodium dodecyl sulfate-polyacrylamide gel electrophoresis (SDS-PAGE) gel and detected by western blotting with anti-WRKY70 polyclonal primary antibody and an anti-rabbit IgG conjugated to horseradish peroxidase secondary antibody (Sigma, A-6667).

### Conductivity measurement assay

For conductivity measurement experiment, control transgenic and *CaWRKY70* overexpressing chickpea roots were subjected to Foc1 infection at various times and washed thoroughly with water. 200 milligram control and Foc1 infected chickpea roots were incubated overnight in sterile tubes filled with 20.0 ml distilled water. Following this, electrical conductivity of water was measured using an electrolyte meter at indicated time points.

### Estimation of Foc1 biomass

Amount of Foc1 biomass was measured according to the previously described method [76]. Genomic DNA isolated from Foc1 inoculated root tissues of control transgenic and *CaWRKY70* over-accumulating chickpea was used as template for real-time PCR with 5.8S rDNA primers listed in (Additional file 3: Table S2).

### Measurement of relative water content (RWC)

RWC of vehicle transgenic and *CaWRKY70* overexpressing chickpea plants were determined by weighing method upon control treatment and Foc1 infection [38].

### SA estimation by high performance liquid chromatography (HPLC)

SA concentrations were determined by HPLC (Shimadzu, Japan) provided with two LC-10 pumps and a UV detector system SPD-10A [77]. Total SA was extracted from 200 mg control and Foc1 treated shoot tissues of transgenic chickpea. The samples were dissolved separately in 200 μl of running buffer (0.2 M NaOAc, pH 5.2, and 10% methanol) and injected in a C-18 HPLC column (4 μm, 250 × 4.6 mm, Phenomenex, USA). A two-pump linear gradient system was used for separation of methanolic plant extracts i.e., pump A contains 1% acetic acid and pump B was filled with acetonitrile. SA detection was carried out at 254 nm wavelength, 30 °C temperature with a flow rate of 0.8 ml/min. The data obtained were combined using Shimadzu Class VP series software. Samples were identified according to their respective retention time (Rt) of peaks and quantity was calculated in mg g^− 1^ FW based on area of the peak and the values obtained for standard used.

### In silico DNA-protein interaction study

The sequence of putative CaWRKY70 protein was retrieved from NCBI (National Centre for Biotechnology Information). Template search and three-dimensional structure prediction of CaWRKY70 (PDB ID: c2aydA) was performed by homology modelling using Phyre2 tool (http://www.sbg.bio.ic.ac.uk/phyre2) [78]. Validation of the generated model was further carried out by Ramachandran plot analysis using RAMPAGE server (http://mordred.bioc.cam.ac.uk/~rapper/rampage.php) [79]. Molecular docking analysis of CaWRKY70 with W-Box DNA was done by HADDOCK (High Ambiguity Driven protein-protein Docking, www.haddocking.org) web server [80]. B DNA model with W-Box element (TGAC) was constructed using 3D-DART webserver, which corrects the nucleotide according HADDOCK specification [81]. Protein and DNA models were subjected to molecular docking by uploading their respective PDB files using easy interface at the HADDOCK server. The W-Box element of modelled DNA and the “WRKYGQK” amino acid sequences present in CaWRKY70 transcription factor were selected as the active residues for docking. Passive residues were automatically selected surrounding the active residues. Finally, docked structure was illustrated and visualized using UCSF Chimera (https://www.cgl.ucsf.edu/chimera/) [82].

### Chromatin immunoprecipitation (ChIP) assay

ChIP assay was performed according to the method previously described by [83] showing CaWRKY70 and CaWRKY40 binding at *CaWRKY40* and *CaMPK9* promoters via W-boxes, respectively. ChIP primers are listed in the (Additional file 3: Table S2).

### Electrophoretic mobility shift assay (EMSA)

In vitro DNA binding activity of 6X histidine tagged WRKY70 protein was performed using EMSA experiments. To prepare the DNA probes for EMSA, equimolar concentration of each sense and antisense oligonucleotide of respective DNA duplexes were mixed in a reaction buffer containing 40 mM Tris−HCl; pH 7.5, 20 mM MgCl_2_, 50 mM NaCl. The reaction mix was heated at 95 °C for 10 min and slowly cooled down to room temperature for annealing. DNA duplexes were run on 7% PAGE (polyacrylamide gel electrophoresis) and gel-purified, followed by end labelling with γ-^32^P and T_4_-polynucleotide kinase (NEB). The labeled probes were then purified using QIAquick Nucleotide Removal Kit (Qiagen). The binding assay was performed using His-tagged WRKY70 protein. Typically, the binding reaction contained 100–200 ng of purified protein, 10 ng of double-stranded synthetic oligonucleotides end-labeled with γ-^32^P in a binding buffer containing 20 mM HEPES, pH 7.5, 100 mM KCl, 0.2 mM EDTA, 1 mM DTT, 2 mM MgCl_2_, and 1 μg of poly dI-dC. The protein was pre-incubated in binding buffer for 5 min prior to the addition of probe for eliminating the risk of non-specific binding and the reaction mix was incubated for another 30 min at room temperature. The complexes were resolved in 0.5X TBE-5% PAGE at (4 °C, 100 V) for about 2 h. After electrophoresis, gels were dried and exposed to phosphorscreen for imaging in a phosphorimager (Typhoon, GE Healthcare). For competition assays, unlabelled duplexes were added during the incubation stage.

### Protoplast transfection and trans-inhibition assay

Protoplasts from *Nicotiana tabacum* cv. Xanthi (Brad) cell suspension culture was isolated and electroporated according to previously described method by [84]. For trans-inhibition assay, *CaWRKY40* promoter region was cloned between *Hind*III/ *BamH*I site by replacing CaMV35S promoter in YFP containing pBI121 vector to obtain *pWRKY40*:*YFP*. Next, *p35S:WRKY70* and *pWRKY40*:*YFP* vectors were co-transfected into protoplasts and incubated in dark for 48 h. Confocal microscopy was performed to monitor the promoter activity.

### GUS assay

Histochemical GUS staining of transgenic tobacco seedlings were performed according to the protocol [85]. Briefly, the tissues were incubated in GUS staining solution containing 1 mM X-Gluc (Duchefa Biochemie, Netherlands), 100 mM sodium phosphate (pH 7.0), 2 mM potassium ferricyanide, 2 mM potassium ferrocyanide, 10 mM EDTA, and 0.1% Triton X-100 under dark conditions at 37 °C for overnight (16 h). After staining, tissues were de-stained in 75% ethanol and photographed with a digital camera. *CaWRKY40* (LOC101512877) and *CaMPK9* (LOC101496681) promoter activity was monitored through the GUS expression analysis. *CaWRKY40* and *CaMPK9* promoter was inserted between *Hind*III/ *Bam*HI site of pBI121 vector by replacing CaMV35S promoter upstream to the *GUS* gene. Plasmids were transformed into *A. tumefaciens* strain LBA4404. *p35S:WRKY70* or *p35S:WRKY40* (effector constructs) and *pWRKY40:GUS* or *pCaMPK9:GUS* (reporter constructs) were co-infiltrated into the ventral surface of *N. tabacum* leaves and subjected to GUS activity assay at 2 days post-infiltration. 100 milligram of agro-infiltrated tobacco leaf discs were collected in a 1.5 ml micro-centrifuge tube and crushed with liquid nitrogen into fine powder. Five hundred microliter of GUS extraction buffer containing 50 mM NaHPO4, pH- 7.0, 10 mM 2-mercapto ethanol, 10 mM Na_2_EDTA, 0.1% SDS and 0.1% triton X-100 was added to grinded sample. The mixture was then centrifuged at 13,000 rpm for 15 min at 4 °C and the cleared supernatant was collected. Data was normalized to protein concentration as measured by Bradford method [75]. Ten microgram crude protein extracts were mixed with 100 μl of GUS assay solution 2 mM 4-methyl umbelliferyl-d-glucuronide (4-MU) in extraction buffer. The reaction was carried out at 37 °C for 60 min and stopped by addition of 0.2 M Na_2_CO3. One hundred microliter reaction mixture was used to measure the fluorescence of GUS enzymatic activity using spectro-fluorometer (Hitachi, F-7000) under excitation and emission at 365 nm 455 nm.

### *Agrobacterium* mediated transient infiltration

*Agrobacterium tumefaciens* GV3101 cells harbouring plasmids were grown overnight at 28 °C in 25 ml Luria-Bertani-broth with antibiotics. Next day, bacterial cells were pelleted by centrifugation at 8000 rpm and dissolved in 20 ml of induction medium (50 mM MES, pH 5.6, 0.5% (W/V) Glucose, 1.7 mM NaH_2_PO_4_, 20 mM NH_4_Cl, 1.2 mM MgSO_4_, 2 mM KCl, 17 μM FeSO_4_ and 70 μM CaCl_2_) in presence of 200 μM acetosyringone. The solution was incubated at 28 °C for 4 h in shaker incubator with rotation speed 160 rpm. After incubation, bacterial cells were pelleted, resuspended in 20 ml of agroinfiltration medium (10 mM MES and 10 mM MgCl_2_, pH 5.6) for 3 h with 100 μM acetosyringone at OD_600_ (0.4) and then infiltrated into the ventral surface of fully expanded 6-weeks-old *N. benthamiana* leaves. In case of chickpea leaves, transient infiltration was carried out using bath-sonication method as previously described by [30].

### BiFC assay

For BiFC studies, full length CDS of the *CaWRKY70* and *CaRPP2*-like *CC-NB-ARC-LRR* gene (GenBank accession XM_012712097.1) was cloned between *BamH*I/ *Sal*I site of pSPYCE and pSPYNE vectors to generate *Ca*WRKY70-YFP^C-ter.^ and CC-NB-ARC-LRR-YFP^N-ter.^. Full-length *CaWRKY64* gene (GenBank accession XM_004489016.3) was cloned within *Spe*I/ *Xho*I site of pSPYCE vector. The control and fusion plasmids were transformed into *Agrobacterium* strain GV3101. *Agrobacterium* cells carrying control and fusion plasmids were co-infiltrated into the abaxial-side of *Nicotiana benthamiana* leaves by *Agrobacterium* mediated transient transformation according to the previously described method [30]. After 48 h post-infiltration, epidermal cells were peeled off and subjected to confocal microscopy for reconstitution of the YFP signal.

### Co-immunoprecipitation assay

For co-IP experiment, c-myc epitope tagged CC-NB-ARC-LRR protein and WRKY70 were transiently co-expressed in *N. benthamiana* leaves by *Agrobacterium* strain GV3101. At 2 dpi, 1 g of leaf samples were collected and quickly frozen in liquid nitrogen. Total protein was extracted from the infiltrated leaves using 2.5 mL ice cold protein extraction buffer containing 50 mM HEPES pH -7.5, 150 mM NaCl, 400 mM sucrose, 10% glycerol, 10 mM EDTA, 1% (v/v) Nonidet P-40, 0.5% (w/v) sodium deoxycholate and protease inhibitor cocktail (Cat# 9599, Sigma-Aldrich, St Louis, Mo, USA). Crude protein extracts were centrifuged at 14,000 rpm for 20 min at 4 °C. The supernatants were pre-cleared using protein A-agarose beads and incubated with 10 μl of anti-Myc (ab39688) or anti-WRKY70 antibody for 3 h at 4 °C in a rotary shaker. One hundred microliter protein A-agarose beads (Bio-Bharati life science) were added to the samples and incubated for additional 3 h at 4 °C. The bound proteins were separated by centrifugation at 14,000 rpm for 20 min at 4 °C. Next, the beads were washed three times with ice cold wash buffer (50 mM HEPES pH 7.5, 150 mM NaCl, 10 mM EDTA and 0.1% Triton X-100 and protease inhibitor cocktail). Bound proteins were eluted from the beads by 100 μl 1X laemmli sample buffer containing 250 mM Tris-HCl (pH 6.8), 10% SDS (W/V), 0.5% bromophenol blue (W/V), 50% glycerol and 50 mM DTT in a boiling water bath for 10 min. Western blotting was performed with anti-c-Myc and anti-WRKY70 antibodies, which is followed by addition of the horseradish peroxidase conjugated secondary antibody.

### Statistical analyses

For statistical differences, Student’s *t*-test was performed at a significance level of *p* < 0.05 come next to multiple comparison of means by Tukey’s post-hoc test.

## Supplementary information

**Additional file 1 Figure S1.** Subcellular localization of control YFP and CaWRKY70-YFP after transient expression in onion epidermal cells by *Agrobacterium*. Bars represent 250 μm. Red and white arrows indicate nucleus and cytoplasm, respectively. **Figure S2.** PCR amplification of *CaWRKY70* gene cloned in pCAMBIA2301 vector after resolved on 1.2% agarose gel. Lane-M denotes DNA molecular weight marker in kilobases (kb). Lane-1, 2, and 3 show positive clones. Red arrows indicate *CaWRKY70* PCR amplicons. **Figure S3.** Homology modelling and Ramachandran plot calculation of CaWRKY70 protein. **a**, **b** and **c** Predicted structure of CaWRKY70 showing five anti-parallel β-strands. **d**, **e** Qualitative assessment of stereo chemical and spatial arrangement of amino acids present on CaWRKY70 protein using RAMPAGE server. **Figure S4.** MUG assay quantitation of CaWRKY70 mediated reduction in *pWRKY40*-GUS activity. Plus (+) and minus (−) signs show presence or absence of the specific components. Error bars represent ±SD (*n* = 5). Asterisks (*) indicate values are different from one another in statistically significant manner as determined by Student’s *t* test (*** *P* < 0.001). **Figure S5.***CaMPK9* transcript accumulation in susceptible JG62 and resistant WR315 chickpea shoots under control treatment (0 dpi) and Foc1 infection (7 dpi) by real-time PCR. *CaGAPDH* mRNA level was used as internal control. Fold change was calculated relative to the control treatment. Error bars indicate ±SD of three biological replicates. Student’s *t* test was performed to determine its significance level as compared to the control treatment, **P ≤ 0.01 and ***P ≤ 0.001.

**Additional file 2 Table S1.** Statistics of the top five clusters of CaWRKY70-DNA complex generated by HADDOCK server.

**Additional file 3 Table S2.** List of primers used in this study.

## Data Availability

No large-scale global data or any database was created for the study. All data generated during this study are included in this article and additional files.

## Abbreviations

ABA: Abscisic acid
ARC: Apaf-1, Resistance and CED4 (*Caenorhabditis elegans* cell death 4 protein)
BiFC: Bimolecular Fluorescence Complementation
Ca: *Cicer arietinum* L.
CBB: Coomassie Brilliant Blue
CC: Coiled coil
ChIP: Chromatin Immunoprecipitation
DNA: Deoxyribonucleic acid
EDS1: Enhanced Disease Susceptibility 1
EMSA: Electrophoretic Mobility Shift Assay
ET: Ethylene
HA: Haemagglutinin
HR: Hypersensitive response
ICS1: Isochorismate Synthase 1
JA: Jasmonic acid
LRR: Leucine rich repeat
MPK: Mitogen Activated Protein Kinase
mRNA: Messenger RNA
NB: Nucleotide binding
NLR: Nucleotide binding oligomerization domain leucine rich repeat
NPR1: Non-expressor of PR1
PAD4: Phytoalexin Deficient 4
PAGE: Polyacrylamide Gel Electrophoresis
PAL: Phenylalanine ammonia-lyase
PCR: Polymerase chain reaction
PDB: Protein Data Bank
PR1: Pathogenesis Related 1
qRT-PCR: Quantitative real-time PCR
RNA: Ribonucleic acid
RPP2: Recognition of *Peronospora Parasitica* 2
SA: Salicylic acid
SDS: Sodium Dodecyl Sulfate
YFP: Yellow Fluorescent Protein
