# Supplementary material for: Inhibition of multiple defense responsive pathways by CaWRKY70 transcription factor promotes susceptibility in chickpea under Fusarium oxysporum stress condition
Source: BMC Plant Biol. 2020 Jul 6;20:319. doi: 10.1186/s12870-020-02527-9 (PMC7336453; doi:10.1186/s12870-020-02527-9)
Supplement: Supplementary file 1 — Additional file 1 Figure S1. Subcellular localization of control YFP and CaWRKY70-YFP after transient expression in onion epidermal cells by Agrobacterium. Bars represent 250 μm. Red and white arrows indicate nucleus and cytoplasm, respectively. Figure S2. PCR amplification of CaWRKY70 gene cloned in pCAMBIA2301 vector after resolved on 1.2% agarose gel. Lane-M denotes DNA molecular weight marker in kilobases (kb). Lane-1, 2, and 3 show positive clones. Red arrows indicate CaWRKY70 PCR amplicons. Figure S3. Homology modelling and Ramachandran plot calculation of CaWRKY70 protein. a, b and c Predicted structure of CaWRKY70 showing five anti-parallel β-strands. d, e Qualitative assessment of stereo chemical and spatial arrangement of amino acids present on CaWRKY70 protein using RAMPAGE server. Figure S4. MUG assay quantitation of CaWRKY70 mediated reduction in pWRKY40-GUS activity. Plus (+) and minus (−) signs show presence or absence of the specific components. Error bars represent ±SD (n = 5). Asterisks (*) indicate values are different from one another in statistically significant manner as determined by Student’s t test (*** P < 0.001). Figure S5.CaMPK9 transcript accumulation in susceptible JG62 and resistant WR315 chickpea shoots under control treatment (0 dpi) and Foc1 infection (7 dpi) by real-time PCR. CaGAPDH mRNA level was used as internal control. Fold change was calculated relative to the control treatment. Error bars indicate ±SD of three biological replicates. Student’s t test was performed to determine its significance level as compared to the control treatment, **P ≤ 0.01 and ***P ≤ 0.001. [file 12870_2020_2527_MOESM1_ESM.pdf]

Supplementary material

Title

Inhibition of multiple defense responsive pathways by CaWRKY70 transcription factor promotes susceptibility in chickpea under *Fusarium oxysporum* stress condition

Authors

Joydeep Chakraborty[1], Senjuti Sen[1], Prithwi Ghosh[1, 2], Akansha Jain[1], Sampa Das[1]*

Affiliations

[1]Present Address

Division of Plant Biology, Bose Institute, Centenary Campus, P-1/12, CIT Scheme-VIIM, Kankurgachi, Kolkata 700054, West Bengal, India

[2]Present Address

Institute of Biological Chemistry, Washington State University, Pullman, Washington, United States of America

*****Corresponding Author: Phone: 91-33-25693251; Fax: 91-33-23553886

Prof. Sampa Das (sampa@jcbose.ac.in)

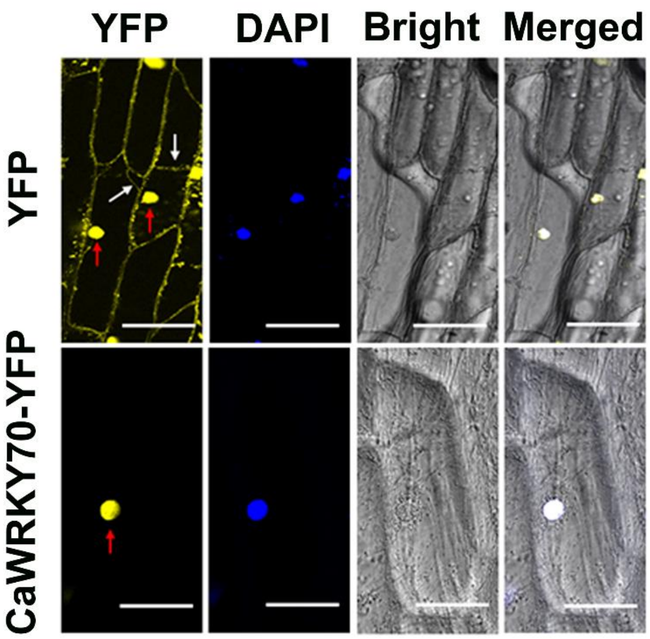

Fig. S1. Subcellular localization of control YFP and CaWRKY70-YFP after transient expression in onion epidermal cells by *Agrobacterium*. Bars represent 250 μm. Red and white arrows indicate nucleus and cytoplasm, respectively.

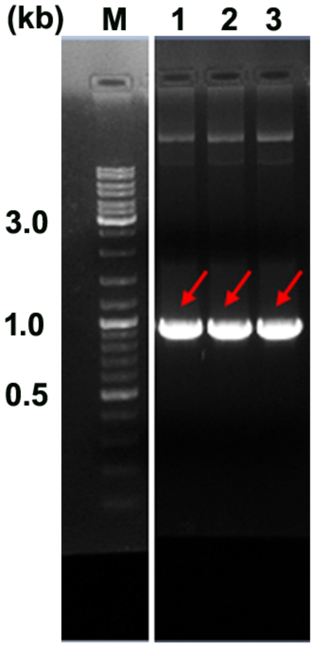

Fig. S2. PCR amplification of *CaWRKY70* gene cloned in pCAMBIA2301 vector after resolved on 1.2% agarose gel. Lane-M denotes DNA molecular weight marker in kilobases (kb). Lane-1, 2, and 3 show positive clones. Red arrows indicate *CaWRKY70* PCR amplicons.

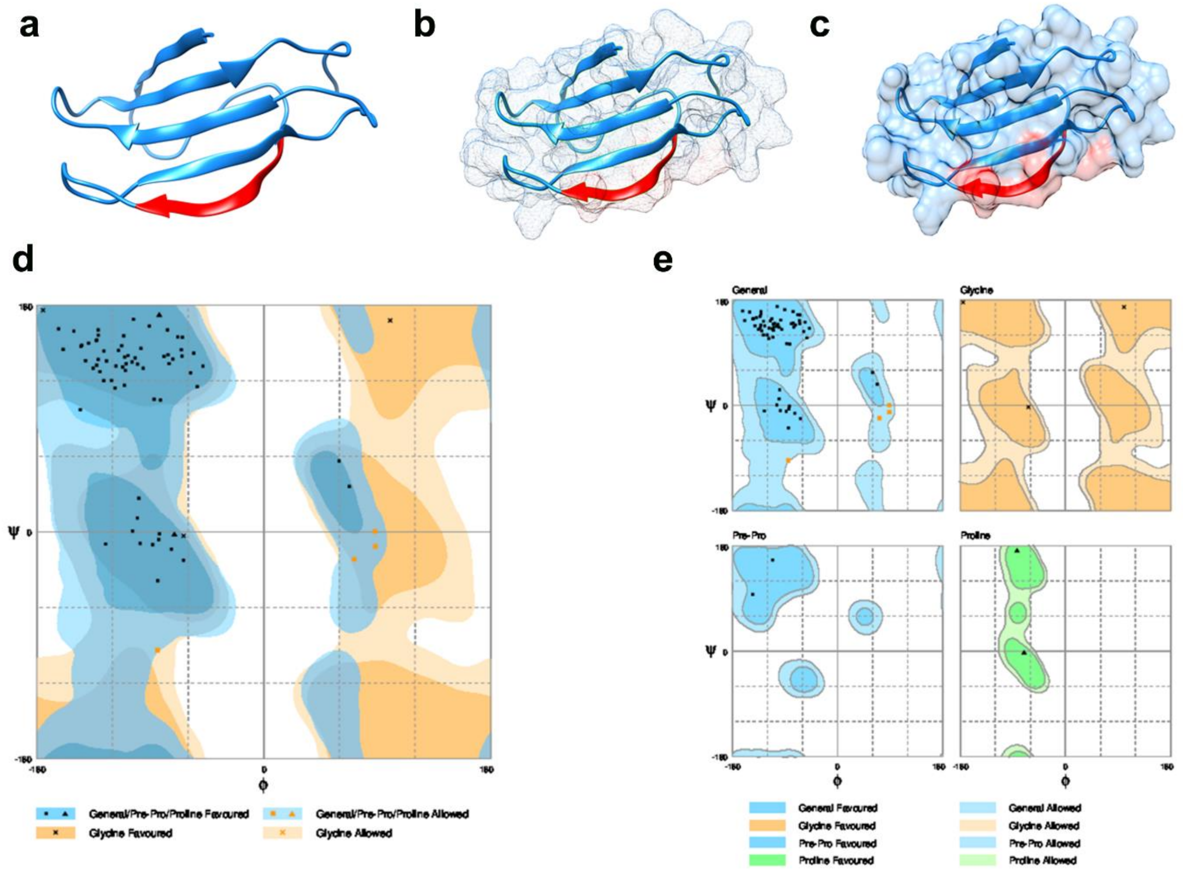

Fig. S3. Homology modelling and Ramachandran plot calculation of CaWRKY70 protein. **a**, **b** and **c** Predicted structure of CaWRKY70 showing five anti-parallel β-strands. **d**, **e** Qualitative assessment of stereo chemical and spatial arrangement of amino acids present on CaWRKY70 protein using RAMPAGE server.

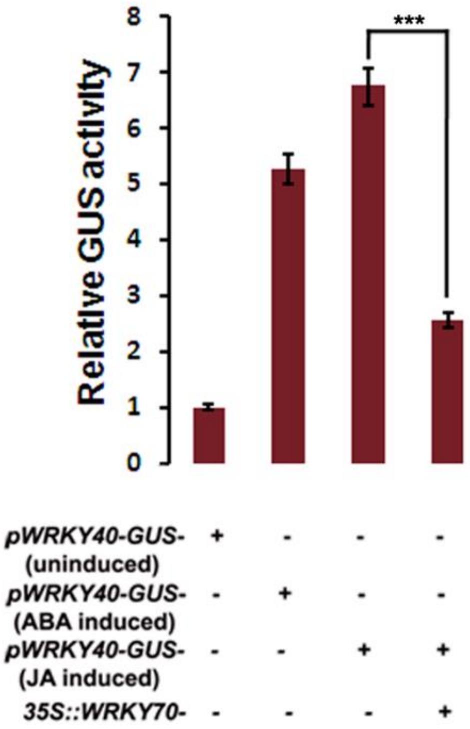

Fig. S4. MUG assay quantitation of CaWRKY70 mediated reduction in *pWRKY40*-GUS activity. Plus (+) and minus (-) signs show presence or absence of the specific components. Error bars represent ±SD (n = 5). Asterisks (*) indicate values are different from one another in statistically significant manner as determined by Student's *t* test (*** $P < 0.001$).

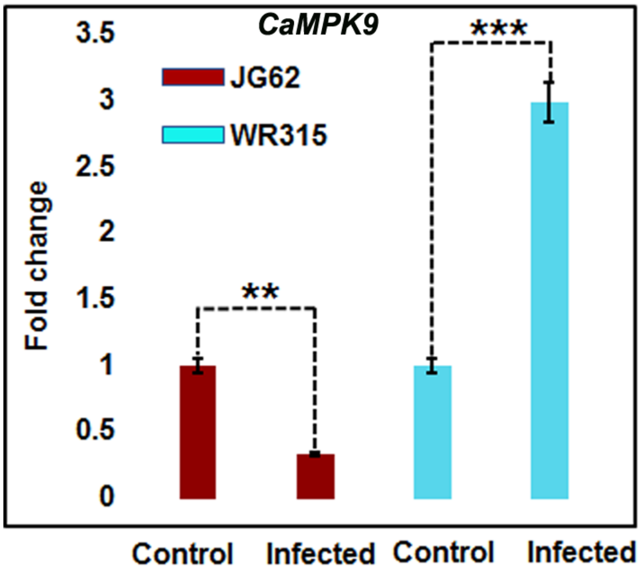

Fig. S5. *CaMPK9* transcript accumulation in susceptible JG62 and resistant WR315 chickpea shoots under control treatment (0 dpi) and Foc1 infection (7 dpi) by real-time PCR. *CaGAPDH* mRNA level was used as internal control. Fold change was calculated relative to the control treatment. Error bars indicate ±SD of three biological replicates. Student's *t* test was performed to determine its significance level as compared to the control treatment, $^{**}P \leq 0.01$ and $^{***}P \leq 0.001$.
