# Supplementary material for: Inhibition of multiple defense responsive pathways by CaWRKY70 transcription factor promotes susceptibility in chickpea under Fusarium oxysporum stress condition
Source: BMC Plant Biol. 2020 Jul 6;20:319. doi: 10.1186/s12870-020-02527-9 (PMC7336453; doi:10.1186/s12870-020-02527-9)
Supplement: Supplementary file 2 — Additional file 2 Table S1. Statistics of the top five clusters of CaWRKY70-DNA complex generated by HADDOCK server. [file 12870_2020_2527_MOESM2_ESM.pdf]

Supplementary Table S1 Statistics of the top five clusters of CaWRKY70-DNA complex generated by HADDOCK server

Models	HADDOCK score (a.u)	RMSD (Å)	Van der Waals energy (Kcal/mol)	Electrostatic energy (J)	Desolvation energy (kcal/mol)	Restraints violation energy (kcal/mol)	Buried Surface Area (Å^2)	Z-Score
Cluster 3	-128.8 +/- 2.5	1.9 +/- 1.3	-49.4 +/- 1.9	-477.9 +/- 24.0	14.1 +/- 3.2	20.3 +/- 16.87	1248.2 +/- 74.0	-2.5
Cluster 12	-104.5 +/- 9.4	14.8 +/- 0.1	-42.7+/-2.1	-408.0 +/- 46.7	18.8 +/- 1.7	10.1 +/- 14.21	1136.6 +/- 92.0	-0.6
Cluster 1	-101.8 +/- 3.8	15.2 +/- 0.1	-36.6 +/- 2.2	-376.4 +/- 31.4	9.7 +/- 2.6	3.3 +/- 2.04	972.1 +/- 47.2	-0.5
Cluster 2	-100.4 +/- 2.0	15.3 +/- 0.0	-38.6 +/- 2.7	-388.3 +/- 16.9	13.8 +/- 1.6	21.4 +/- 20.62	1054.3 +/- 31.6	-0.3
Cluster 5	-94.4 +/- 6.3	3.2 +/- 0.3	-44.3 +/- 2.3	-351.1 +/- 28.8	16.4 +/- 3.2	36.8 +/- 1.69	1022.6 +/- 102.9	0.1
