# Supplementary material for: Inhibition of multiple defense responsive pathways by CaWRKY70 transcription factor promotes susceptibility in chickpea under Fusarium oxysporum stress condition
Source: BMC Plant Biol. 2020 Jul 6;20:319. doi: 10.1186/s12870-020-02527-9 (PMC7336453; doi:10.1186/s12870-020-02527-9)
Supplement: Supplementary file 3 — Additional file 3 Table S2. List of primers used in this study. [file 12870_2020_2527_MOESM3_ESM.pdf]

Supplementary Table S2 List of primers used in this study

Primers used for real-time PCR experiment

Primer name	Oligonucleotide sequence (5’ to 3’)
CaWRKY70-F	TCATGGATCTCCCTCGGTTA
CaWRKY70-R	CTTCGATCCTTTGTGGTGGT
CaWRKY33-F	TGGTTTTGGTGATCGTGGTG
CaWRKY33-R	CACCAGTAGTAGGAGATGGCA
CaWRKY54-F	GGGTCCTCTTCTTTCCCTTG
CaWRKY54-R	TGGTGGTTCAACACCTCAGA
CaWRKY40-F	GAGCCTTGGTTCGGTACCTT
CaWRKY40-R	TTCCTGATATGGCTGCAACA
CaMPK9-F	ATGTGATTAAAATTAAGGAC
CaMPK9-R	GCGTTTGGAGGGATCAAAAA
CaICS1-F	CCTAATGCACCGGCATTTAT
CaICS1-R	GACCATTTTCTTGGGCTTGA
CaPAL-F	TGTTTGCAATGGAAATGGAA
CaPAL-R	AGCCTTAACCGCATATCGTG
CaEDS1-F	TTTGGTTCTCCCTTGATTGG
CaEDS1-R	TCCCACATTCCTCATCACAA
CaPAD4-F	AGAGCCAAATGGGTCAACAC
CaPAD4-R	CCCAAAAGCACGAATCTTGT
CaNPR1-F	AAGCGTGGGAAAGTGAATTG
CaNPR1-R	CCCACTTGCTGCAAAGAAAT
CaPR1-F	CTTCAAAATGCACTATAGAC
CaPR1-R	TCCGTTGAGAAAACTTTGGTT
CaTGA1-F	ACAAGGCATGGACAAACTCC
CaTGA1-R	ACATTTGCTGCAAGGTTTCC
CaTGA6-F	ATATGCAAGGTGGCTGGAAG
CaTGA6-R	CTCAGCTGGTGTTTTCCACA
CaPR5-F	AGGGGAAGACTGGCATCACT
CaPR5-R	CAGCACCACTTCCATCACTC
CaDefensin-F	AAAAAGCTTAGTCGAGTGAGATGAAT
CaDefensin-R	AAAGGATCCGGCTTAGTGATATTGA
CaGAPDH-F	CATTGTTCACATTGCCAAGC
CaGAPDH-R	CCAAAACAGCTCATTGCTCA
Foc1 5.8S rDNA-F	GTTGAAATGACGCTCGAACA
Foc1 5.8S rDNA-R	GCCAGAGGACCCCTAAACTC

Primers used for *CaWRKY70* gene cloning in pCAMBIA2301 vector

Primer name	Oligonucleotide sequence (5’ to 3’)
pCAMBIA*CaWRKY70*-F	AGTCGGATCCATGGAGAATCTATTTGGG
pCAMBIA*CaWRKY70*-R	ATTACTCGAGAATCAAATGACTTTCATCAA

Primers used for *CaWRKY40* gene cloning in pCAMBIA2301 vector

Primer name	Oligonucleotide sequence (5' to 3')
pCAMBIA*CaWRKY40*-F	AATGGATCCATGGATTGTTCATCATATAT
pCAMBIA*CaWRKY40*-R	AATCTCGAGATTATTGTGCATCAATCTTC

Primers used for *CaWRKY70* gene cloning in pBI-YFP vector

Primer name	Oligonucleotide sequence (5' to 3')
pBI-*CaWRKY70*-F	ATGTGGATCCATGGAGAATCTATTTGGG
pBI-*CaWRKY70*-R	AAGAGAGCTCAATCAAATGACTTTCATCAA

Primers used for bacterial expression study

Primer name	Oligonucleotide sequence (5' to 3')
pET*CaWRKY70*-F	AATGAATTCATGGAGAATCTATTTGGG
pET*CaWRKY70*-R	AATCTCGAGAATCAAATGACTTTCATCAA
pET*CaWRKY40*-F	ATAGAATTCATGGATTGTTCATCATATA
pET*CaWRKY40*-R	AATCTCGAGATTATTGTGCATCAATCTT

Primers used for cloning in BiFC vectors

Primer name	Oligonucleotide sequence (5' to 3')
pSPYNE*CC-NLR*-F	AATTGGATCCCTGATGTCCCTTACTGTTGATTT
pSPYNE*CC-NLR*- R	AATTGTCGACCTAAATCAATTTGTCGTCAA
pSPYCE*CaWRKY64*-F	AATTACTAGTATGGAGAATCTATTTGGGCTT
pSPYCE*CaWRKY64*-R	AATTCTCGAGCTAAATCAATTTGTCGTCAA
pSPYCE*CaWRKY70*-F	ATTAGGATCCATGGAGAATCTATTTGGG
pSPYCE*CaWRKY70*-R	ATTGGTCGACAATCAAATGACTTTCATCAA
pSPYNE*NB-ARC*- F	AATTGGATCCATGTCCCTTACTGTT
pSPYNE*NB-ARC*- R	AATTGTCGACCTTGTTACCGATCTT

Primers used for ChIP assay

Primer name	Oligonucleotide sequence (5' to 3')
*pCaWRKY40*W1-F	AATTAAAGTTCGTTAACTATTT
*pCaWRKY40*W1-R	ATGTGGATTAATGTTATTTGGACC
*pCaWRKY40*W2-F	CATCAAACATAGGTAAATTAAAA
*pCaWRKY40*W2-R	AGATAGCACTTGAGAAACTCAAG
*pCaMPK9*W1-F	GGATGGACCCTGTAACTGTTAT
*pCaMPK9*W1-R	TGAGATCACCAGAGGTTCATGG
*pCaMPK9*W2-F	ACAAATCTTTTTTGTTTCAAAA
*pCaMPK9*W2-R	ATCTTTGAGTATAACATGGCATTG

Primers used for promoter activity study

Primer name	Oligonucleotide sequence (5’ to 3’)
pCaWRKY40-F	AATAAGCTTAATTAAAGTTCGTTAACTATT
pCaWRKY40-R	AATGGATCCTTTTGTGAAATAAAATTTGTAT
pCaMPK9-F	AATCCAAGCTTACTTGTTTCTGATCAA
pCaMPK9-R	AACCTGGATCCCAACTTTCCTCAAGGA
*pCaMPK9*D1-R	AACTTGGATCCTTGTATGTTCTTCGT
*pCaMPK9*D2-R	AAATTGGATCCTGCTTGCTTGGTTTC
